# Reproductive Aging Drives Protein Accumulation in the Uterus and Limits Lifespan in *C*. *elegans*

**DOI:** 10.1371/journal.pgen.1005725

**Published:** 2015-12-11

**Authors:** Stephanie M. Zimmerman, Izumi V. Hinkson, Joshua E. Elias, Stuart K. Kim

**Affiliations:** 1 Department of Genetics, Stanford University, Stanford, California, United States of America; 2 Department of Chemical and Systems Biology, Stanford University, Stanford, California, United States of America; 3 Department of Developmental Biology, Stanford University, Stanford, California, United States of America; The University of North Carolina at Chapel Hill, UNITED STATES

## Abstract

Aging in *Caenorhabditis elegans* is characterized by widespread physiological and molecular changes, but the mechanisms that determine the rate at which these changes occur are not well understood. In this study, we identify a novel link between reproductive aging and somatic aging in *C*. *elegans*. By measuring global age-related changes in the proteome, we identify a previously uncharacterized group of secreted proteins in the adult uterus that dramatically increase in abundance with age. This accumulation is blunted in animals with an extended reproductive period and accelerated in sterile animals lacking a germline. Uterine proteins are not removed in old post-reproductive animals or in young vulvaless worms, indicating that egg-laying is necessary for their rapid removal in wild-type young animals. Together, these results suggest that age-induced infertility contributes to extracellular protein accumulation in the uterus with age. Finally, we show that knocking down multiple age-increased proteins simultaneously extends lifespan. These results provide a mechanistic example of how the cessation of reproduction contributes to detrimental changes in the soma, and demonstrate how the timing of reproductive decline can influence the rate of aging.

## Author Summary

To understand the process of aging at the molecular level in *C*. *elegans*, we measured changes in protein abundance with age, determined whether these age-related protein changes lead to dysfunction in old animals, and have elucidated one of the upstream pathways responsible for these aging changes. We found that egg-laying in young worms permits removal of a novel class of proteins present in the uterus. When the reproductive period ends, the removal of uterine proteins stops, causing them to accumulate to toxic levels. This shows that the timing of reproductive decline influences the rate of somatic aging. The concept that the reproductive period has a direct role in specifying the rate of aging of the soma likely applies to other species as well.

## Introduction

Aging in *C*. *elegans* is characterized by stereotyped physiological changes over the course of its three week lifespan. Animals become infertile early in life, followed by a decline in locomotion and the degeneration of many organs, such as the intestine and the muscle [1–3]. Other tissues overproliferate with age; for example, aged worms have increased cuticle thickness, accumulation of yolk protein in the body cavity, masses in the germline, and ectopic neuronal branching [3–6]. Some of these cases of hypertrophy may be related to unchecked protein production or accumulation. However, the upstream causes of these changes and the factors that determine their timing are not yet well understood.

One way to understand the molecular causes of aging is to use unbiased approaches to profile changes that occur with age, and identify the upstream regulators of these changes. Transciptional profiling has been used to identify genes that change expression during *C*. *elegans* aging at the RNA level [7–9]. This has allowed the identification of transcription factors that bind and regulate these age-regulated genes. Many of these transcription factors themselves change expression with age and can modulate lifespan when their expression is reduced or increased [7, 10–12]. However, the mechanisms that induce changes in transcription factor expression, and therefore determine the rate and timing of their change, are generally not known.

Relative to the aging transcriptome, the proteome of aging animals has been less well characterized. Assessing changes in the aging proteome directly is important because changes in RNA abundance are not always predictive of downstream protein abundance changes [13, 14]. In addition, aging has been shown to involve dysregulated protein homeostasis, including reduced protein synthesis and protein folding capacity, and increased proteome insolubility and protein damage [15–21]. Previous studies of the aging proteome in *C*. *elegans* have identified a large number of proteins that aggregate with age [17–19]. In addition, there are large scale changes in soluble protein abundance in old animals [19, 22]. However, the causes of these changes, the factors influencing their timing, and their effect on lifespan remain unclear.

Here, we identify a novel mechanistic link between reproductive aging and somatic aging in *C*. *elegans*. Using mass spectrometry-based proteomics, we identify a previously uncharacterized group of secreted proteins that localize to the adult uterus and dramatically increase in abundance with age. We show that this accumulation is partially driven by the termination of reproduction, a very early event in the aging process. Finally, we show that knocking down multiple age-increased proteins simultaneously extends lifespan. Our results suggest that the cessation of reproduction contributes to changes in the post-reproductive animal that are detrimental for survival, and indicate that the timing of reproductive decline can influence the rate of somatic aging.

## Results

### Identification of proteins that change abundance during aging

In order to interrogate changes in the proteome with age, we isolated protein from young (day 4) and old (day 13) adult *C*. *elegans* and measured relative protein levels by isotopic labeling by reductive dimethylation and liquid chromatography tandem mass spectrometry [23]. Worms were grown on 5-fluoro-2’-deoxyuridine (FUDR) to inhibit progeny production, and strained through a 40 μm pore nylon mesh each day to remove any contaminating eggs and larvae. We chose day 4 for the young sample to ensure that it would be relatively free from contamination by embryos. Day 13 was chosen as the old sample because worms of that age show clear signs of age-related deterioration [1, 3]; however, a majority of the population is still alive at that time (82±7% surviving; S4 Table).

**Fig 1:**
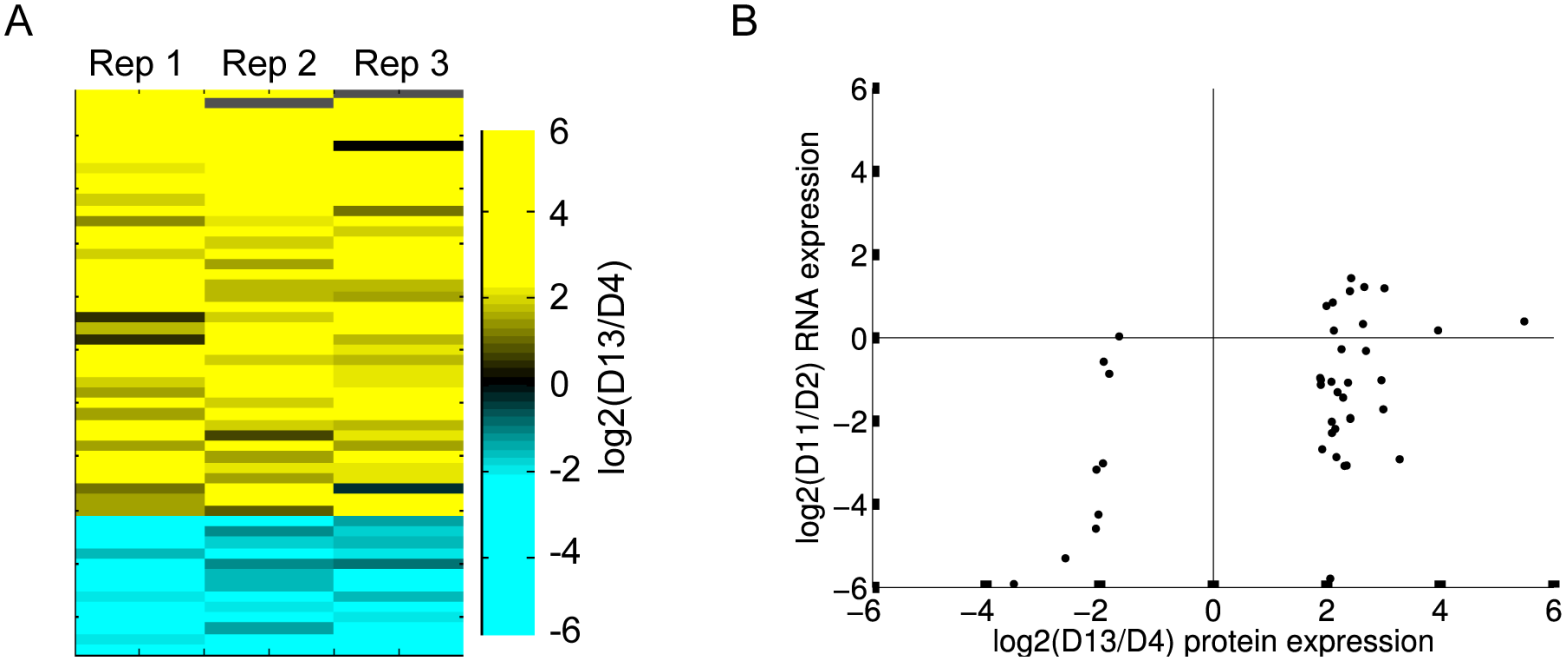
53 proteins change in abundance with age. A. 53 proteins significantly change in abundance with age by rank-product analysis across three biological replicates (10% FDR). Shown is the log_2_ fold change in protein abundance between day 4 and day 13 for the proteins that significantly change. 40 proteins increase abundance with age (yellow), and 13 decrease (blue). B. Proteins that decrease abundance with age are encoded by RNAs that also decrease with age, but there is little correlation between RNA and protein abundance change for proteins that increase abundance with age. The scatterplot shows the log_2_ ratio of old/young protein expression on the x-axis and the log_2_ ratio of old/young RNA expression on the y-axis (microarray expression data from [7]). Each point is a protein that significantly changes in abundance with age and was also covered on the microarray (32 of 40 proteins that increase and 9 of 13 that decrease abundance with age).

We performed three biological replicates of this aging time course. We identified 3159 proteins in total, and 1796 proteins in at least two of the three biological replicates (S1 Table). Of the 1796 proteins that we quantified in at least two replicates, 53 significantly change abundance with age by rank-product analysis at a 10% false discovery rate (FDR) [24]. Forty of these proteins increase in abundance with age, and 13 decrease (Fig 1A, S2 Table).

We used gene set enrichment analysis on the list of 1796 proteins to determine which classes of proteins tended to change with age. Consistent with previous studies of the aging proteome in *C*. *elegans* [19, 22], extracellular proteins were strongly enriched for increasing with age (FDR<10^−4^) and ribosomal proteins were enriched for decreasing with age (FDR<0.01; S3 Table). Furthermore, we found that proteins that increased or decreased in abundance in two previous studies of the aging proteome generally changed in the concordant direction in this study, even if they did not reach our threshold for statistical significance (S1A and S1B Fig).

Previous work also defined a set of proteins that become increasingly insoluble with age [17, 18]. We asked whether loss of solubility might underlie the changes in soluble protein abundance measured in our study. Aggregation of a specific protein in old age could reduce the amount of that protein that is soluble, leading to an apparent decrease in its abundance. However, neither proteins that increased abundance in our study nor those that decreased abundance were enriched for becoming age-insoluble (S1C Fig). None of the 13 proteins that significantly decreased abundance in our data were age-insoluble in both datasets.

Another way to test whether protein aggregation drives changes in the soluble proteome is to ask whether age-insoluble proteins tend to decrease in abundance in the soluble proteome. To do this, we compared the distribution of age-related changes in abundance of insoluble proteins (601 insoluble proteins from [17] and 185 insoluble proteins from [18]) to age-related changes in abundance of all 1796 proteins in our experiment. There was no substantial difference between these two sets, indicating that increasing protein insolubility with age does not substantially deplete the soluble protein pool for most proteins (S1D Fig). This result is consistent with recent findings demonstrating that only a small fraction of the total protein pool becomes age-insoluble for abundant proteins [19].

### Changes in transcriptional regulation explain changes in abundance in old age for most proteins that decrease, but not those that increase

In order to assess underlying mechanisms responsible for changes in protein abundance in old age, we next asked whether proteins that change in abundance with age also show concordant changes in RNA expression during aging. We compared changes in protein abundance of the 53 significantly changed proteins to changes in their respective transcript levels, as measured in a previously generated DNA microarray expression dataset [7] (Fig 1B). We found that proteins that decrease abundance with age also generally decrease at the RNA level. Of the 9 proteins that significantly decreased abundance and were also represented in the microarray dataset, 6 had significantly lower transcript levels with age, a 12-fold enrichment over expectation (p<10^−5^ by Fisher’s exact test). 8 of the 9 proteins had a lower RNA levels regardless of significance. This result suggests that most of our observed decreases in protein abundance in old age can be explained by decreases in their corresponding transcript levels.

However, there was no such concordance for proteins that increased abundance with age. Of the 32 proteins that increase abundance in old age and are also covered in the microarray expression dataset, 14 had significantly decreased RNA expression and just 4 had significantly increased RNA expression with age. This result suggests that most proteins that increase abundance with age in this study do not do so because of corresponding changes in the RNAs that encode them, and instead are likely to increase abundance for a different reason. Thus, we chose to focus our further analysis on the 40 proteins that increase abundance with age.

### Secreted proteins are enriched for increasing abundance with age

**Fig 2:**
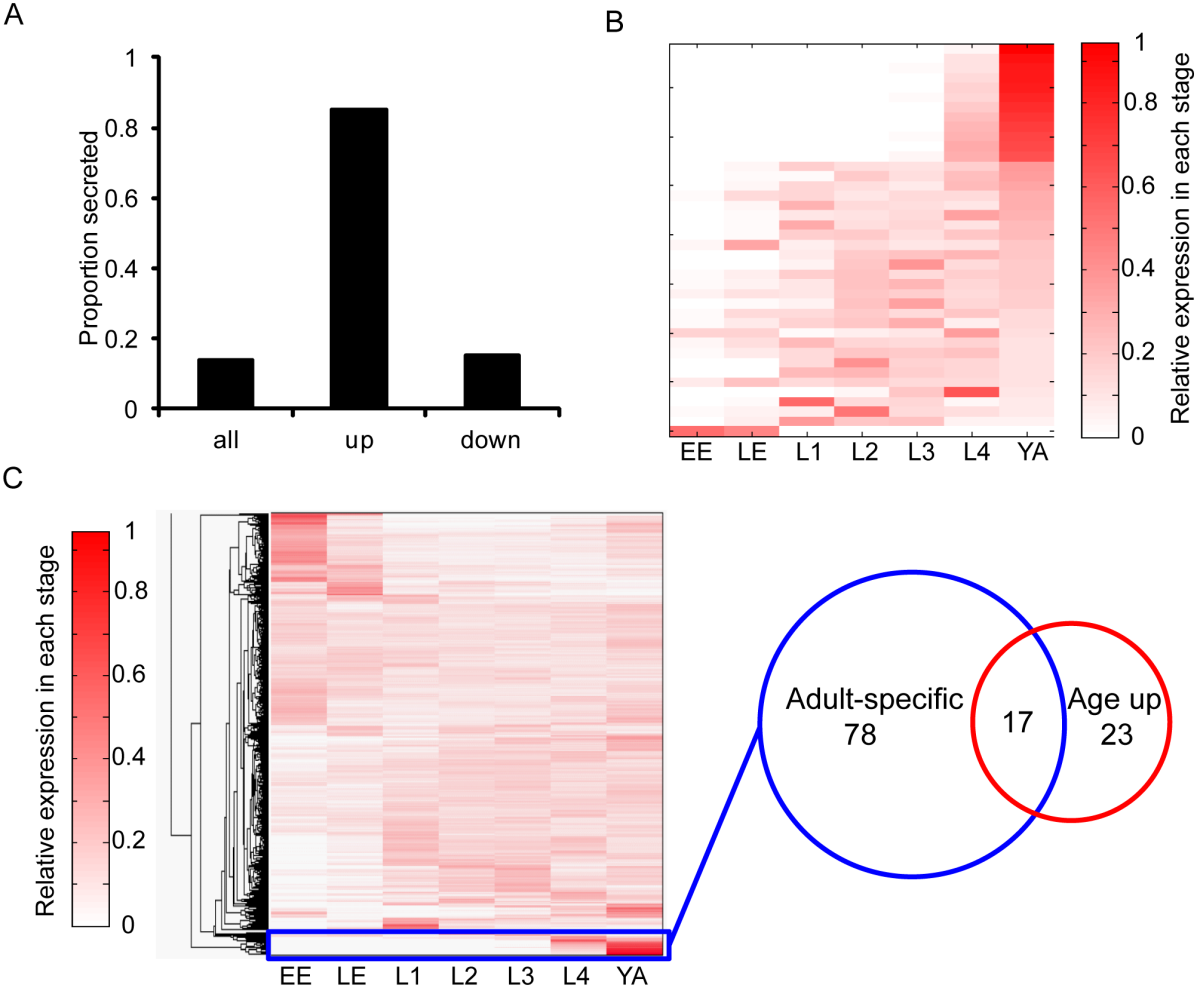
Many proteins that increase in abundance with age are secreted and expressed specifically in adults. A. Proteins that increase in abundance with age are predicted to be secreted (6-fold enrichment over all proteins, p<10^−23^ by Fisher’s exact test). The bar graph shows the proportion of proteins that are predicted to be secreted in the background set of 1796 proteins (all), the 40 proteins that increase in abundance with age (up), and the 13 proteins that decrease with age (down). List of putative secreted proteins from [25]. B. Many proteins that increase in abundance with age are encoded by RNAs expressed specifically in adults. The heat map shows the relative RNA expression in each stage for the 40 proteins that increase in abundance with age. Stage-specific RNA-seq data was generated by the ModEncode consortium [26]. Reads per kilobase per million mapped reads (RPKM) values were normalized across each row to obtain the relative expression of each gene in each stage. EE = early embryo; LE = late embryo; L1–4 = larval stage 1–4; YA = young adult. C. The heat map (left) shows the relative RNA expression in each stage as described above for the 1796 proteins covered in this experiment. The rows are organized by hierarchical clustering. The cluster highlighted by the blue rectangle contains 95 proteins whose RNAs are expressed specifically in young adult worms. The Venn diagram (right) shows the overlap between these 95 proteins (blue circle) and the set of 40 proteins that significantly increase in abundance with age (red circle). 17 proteins are both adult-specific and significantly increase abundance with age (an 8-fold enrichment over expectation, p<10^−11^ by Fisher’s exact test).

Using gene set enrichment analysis, we found that extracellular proteins tend to increase in abundance with age. In total, 249 of the 1796 proteins covered in our experiment (14%) are predicted to be secreted. However, 34 out of 40 (85%) of the proteins that significantly increase abundance with age are putative secreted proteins, a 6-fold enrichment over expectation (p<10^−23^ by Fisher’s exact test; Fig 2A). Furthermore, we compared the distribution of changes in abundance with age for all 1796 proteins covered by our experiment to the 249 proteins that are predicted to be secreted and found that the set of secreted proteins tend to increase abundance with age (p < 10^−27^ by Kolmogorov-Smirnov test; S2A Fig). This trend holds true even for the 215 proteins that remain after filtering out the 34 proteins that are both significantly increased and secreted (p < 10^−17^ by Kolmogorov-Smirnov test; S2A Fig). These results indicate that secreted proteins generally increase abundance during *C*. *elegans* aging, even though many of these individual proteins do not meet our threshold for statistical significance.

### Adult-specific proteins are enriched for increasing abundance with age

To identify possible shared regulation between proteins that increase in abundance with age, we asked whether these proteins are also coexpressed during development. We examined the expression pattern of the transcripts encoding the proteins that increase in abundance with age in early embryos, late embryos, the four larval stages, and day 1 adult worms using data generated by the modEncode consortium [26]. We observed that many of these 40 proteins were expressed specifically in day 1 adults or L4 (late larvae) stage, and not expressed in earlier developmental stages (Fig 2B).

To compare the developmental expression pattern of the proteins that increase abundance with age to all proteins, we performed hierarchical clustering of all 1796 proteins detected in this study on the relative RNA expression in each developmental stage. From this, we identified a cluster that represented transcripts specifically expressed in the day 1 adult (Fig 2C, blue rectangle). In this cluster of 95 proteins, 17 significantly increase in abundance between day 4 and day 13, an 8-fold enrichment over expectation (p<10^−11^ by Fisher’s exact test; Table 1). The observed adult-specific expression pattern is not because these 17 genes are expressed at extremely high levels in adults. Rather, these genes have low RNA expression levels in the embryo and larval stages, and are expressed in young adults at similar levels to that of transcripts encoding the background set of proteins analyzed in this study (S2D Fig).

**Table 1:** 17 age-increased, adult-specific, and secreted proteins.

Name	Sequence ID	Annotation	Aging fold change (log_2_)
*ule-1*	W03F11.1	chitin binding domains	5.5
	T05E12.3		4
*ule-2*	F13G11.3		3.4
*ilys-3*	C45G7.3	invertebrate lysozyme	3
*scl-2*	F49E11.10	sperm coating-like extracellular protein	3
*far-6*	W02A2.2	fatty acid/retinol binding protein	2.7
*ule-3*	D1054.11		2.4
	C39D10.7		2.4
*ule-4*	C08F11.11		2.3
*thn-1*	F28D1.3	thaumatin family	2.3
*ule-5*	Y62H9A.6		2.2
	C36C5.5		2.1
*vit-6*	K07H8.6	vitellogenin	2.1
	C46C2.5		2.1
	D1086.10		2
*cht-3*	C25A8.4	chitinase-like protein	1.9
	F48E3.4	serine-type endopeptidase activity	1.9

We next asked whether adult-specific proteins show a general trend towards increasing abundance with age. The entire group of 95 adult-specific proteins tends to increase with age compared to all 1796 proteins in this study (p < 10^−10^ by Kolmogorov-Smirnov test; S2B Fig). This trend holds true for the 78 proteins that remain after filtering out the 17 proteins that significantly increase in abundance with age (p<10^−4^ by Kolmogorov-Smirnov test; S2B Fig). This suggests that proteins that begin to be expressed on day 1 of adulthood tend to continue to increase in abundance between day 4 and day 13 of adulthood.

Of the 17 proteins that significantly increase in abundance with age and are adult-specific, all are known or predicted to be secreted. Notably, there is a significant overlap between adult-specific proteins and predicted secreted proteins (55 proteins, a 4-fold enrichment over expectation, p < 10^−24^ by Fisher’s exact test). These 55 proteins are strongly shifted towards increasing abundance with age, even though most are below our significance threshold (p < 10^−18^ by Kolmogorov-Smirnov test; S2C Fig). In summary, these results suggest that adult-specific and secreted proteins may define a class of proteins that tend to increase abundance during *C*. *elegans* aging.

### Five secreted, adult-specific, and age-increased proteins localize to the uterus

We hypothesized that this class of secreted, adult-specific, and age-increased proteins might have a common mechanism for their increase in abundance with age and chose to study some members of this group in more detail. Most of 17 proteins in this group have no known molecular function (Table 1). One exception is the yolk protein VIT-6, one of 5 vitellogenin proteins in *C*. *elegans*. Vitellogenins are produced in the intestine, secreted into the body cavity, and taken up by the germ cells in order to provide nourishment for the developing embryos. They are known to accumulate post-reproductively in the body cavity with age [3].

In order to learn more about the uncharacterized members of this group of proteins, we tagged five members with a fluorescent protein at their C-terminus, preserving the entire upstream and downstream elements of the gene (named ULE-1 to ULE-5, as explained below). ULE-3 and ULE-4 were tagged with eGFP, and ULE-1, ULE-2, and ULE-5 were tagged with the photoconvertible fluorescent protein Dendra2.

**Fig 3:**
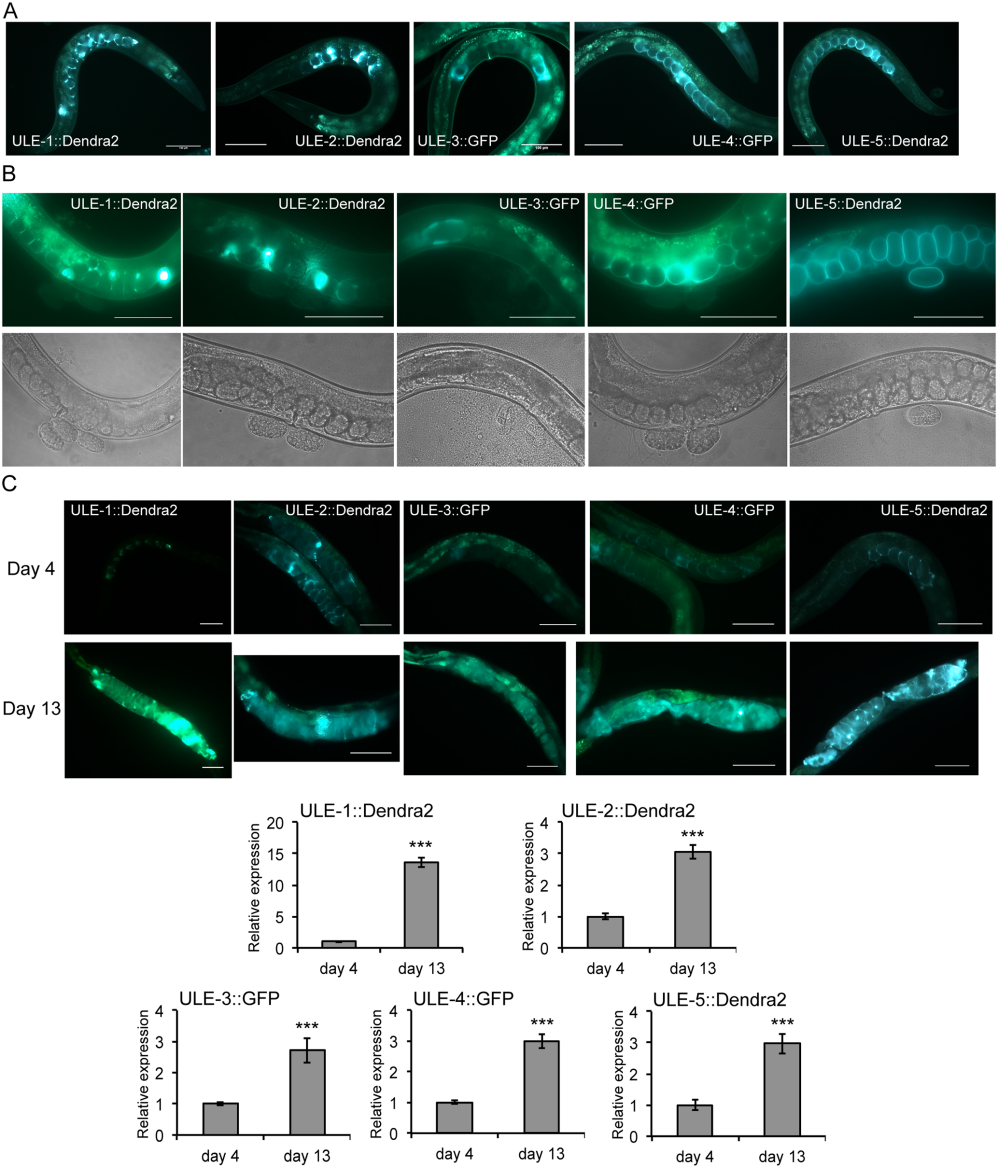
Five age-increased, adult-specific, and secreted proteins are expressed in the uterus. A. Representative images of day 1 adult hermaphrodite worms expressing reporters of five proteins that significantly increase in abundance with age, are predicted to be expressed only in adults, and are predicted to be secreted. All five reporters are expressed exclusively in the uterus around the embryos. Proteins were tagged at the C-terminus with eGFP (ULE-3, ULE-4) or Dendra2 (ULE-1, ULE-2, ULE-5). Scale bar equals 100 microns. B. Representative fluorescent (top) and DIC (bottom) images of a young adult worm that has recently laid eggs for each of the five uterine protein reporter lines. ULE-5::Dendra2 remains associated with the boundary of the egg after it had been laid, but the other four reporters are not visible on or inside embryos outside the mother. Scale bar equals 100 microns. C. Expression of all five uterine protein reporters increase with age. Top: representative images of worms expressing uterine protein reporters at day 4 and day 13 of adulthood. Scale bar equals 100 microns. Bottom: quantification of expression of all five reporters at day 4 and day 13 of adulthood (*** p<0.001 by Student’s t-test for all reporters). The data are represented as mean fluorescent intensity at day 13 relative to mean fluorescent intensity at day 4 (n = ~20 worms in each condition). Error bars are ± SEM.

Surprisingly, we found that all five tagged proteins localized predominantly in the uterus around the embryos (Fig 3A). For four of the five reporters, there was no visible fluorescence associated with the embryo after it had been laid, suggesting that these reporters are localized to the luminal space outside of the embryos within the uterus. For the fifth reporter (ULE-5::Dendra2), fluorescence remained associated with the boundary of the egg after it had been laid, suggesting that this protein is either part of the eggshell or is inside the egg (Fig 3B). Because of the unique expression pattern of these reporters, we named these proteins Uterine Lumen-Expressed (ULE) 1–5.

As expected from their RNA profiles, all five tagged proteins have only weak or absent expression before the L4 stage; ULE-2 and ULE-4 show expression in the region of the developing uterus in L4 animals, while the other three are not visible in this region until adulthood, when embryos are present. Fluorescence expression of the ULE-2, ULE-3, ULE-4 and ULE-5 reporters is limited to the uterus, while ULE-1::Dendra2 is visible in some cells of the hypodermis and tail in approximately 20% of transgenic worms (S3 Fig). There is no visible expression of any of the five uterine protein reporters in young adult male worms.

To verify the proteomic results and identify age-related changes in localization pattern, we examined changes in expression between day 4 and day 13 animals (the same days as used to generate the aging proteome data) for all five uterine protein reporter lines. As expected, all five reporters increase abundance substantially with age (3 to 14-fold; p<0.001 by Student’s t-test for all five reporters). In old worms, the fluorescent reporters completely fill the uterus, which becomes large and distended (Fig 3C). Even in old worms, fluorescent protein expression is confined to the uterus and is not visible in other tissues or within the body cavity.

To confirm that the observed increase in fluorescence was due to reporter expression and not increasing autofluorescence, we treated all five reporter lines with RNAi against the tagged gene and examined the expression at day 4 and day 13. We found that RNAi against each ULE gene decreases expression of its reporter at least 3-fold at both day 4 and day 13 for all five lines (S4B Fig; p<0.001 by Student’s t-test for all five reporters at both timepoints). Furthermore, we observe that autofluorescence in the uterus increases only 30% in non-transgenic control animals between day 4 and day 13 (S4A Fig), suggesting that the 3-14-fold increase in ULE reporter expression that we observe cannot simply be explained by increasing autofluorescence.

Finally, as we used FUDR to inhibit progeny production in the proteomics discovery experiment, we asked whether the use of FUDR affected uterine protein reporter expression with age. We observed a small but significant increase in ULE-2::Dendra2 expression at day 4 (70% increase; p<0.01 by Student’s t-test) and a small but significant decrease in ULE-5::Dendra2 expression at day 13 (20% decrease; p<0.05 by Student’s t-test). There was no change in expression of any of the other reporters at either timepoint. All five reporters increased significantly with age in the absence of FUDR, suggesting that FUDR does not have a substantial effect on uterine protein expression dynamics (S4C Fig). In summary, these results indicate that we have identified a previously uncharacterized group of uterine proteins that dramatically accumulate with age.

### Uterine proteins are secreted by the cells of the uterus and spermatheca

**Fig 4:**
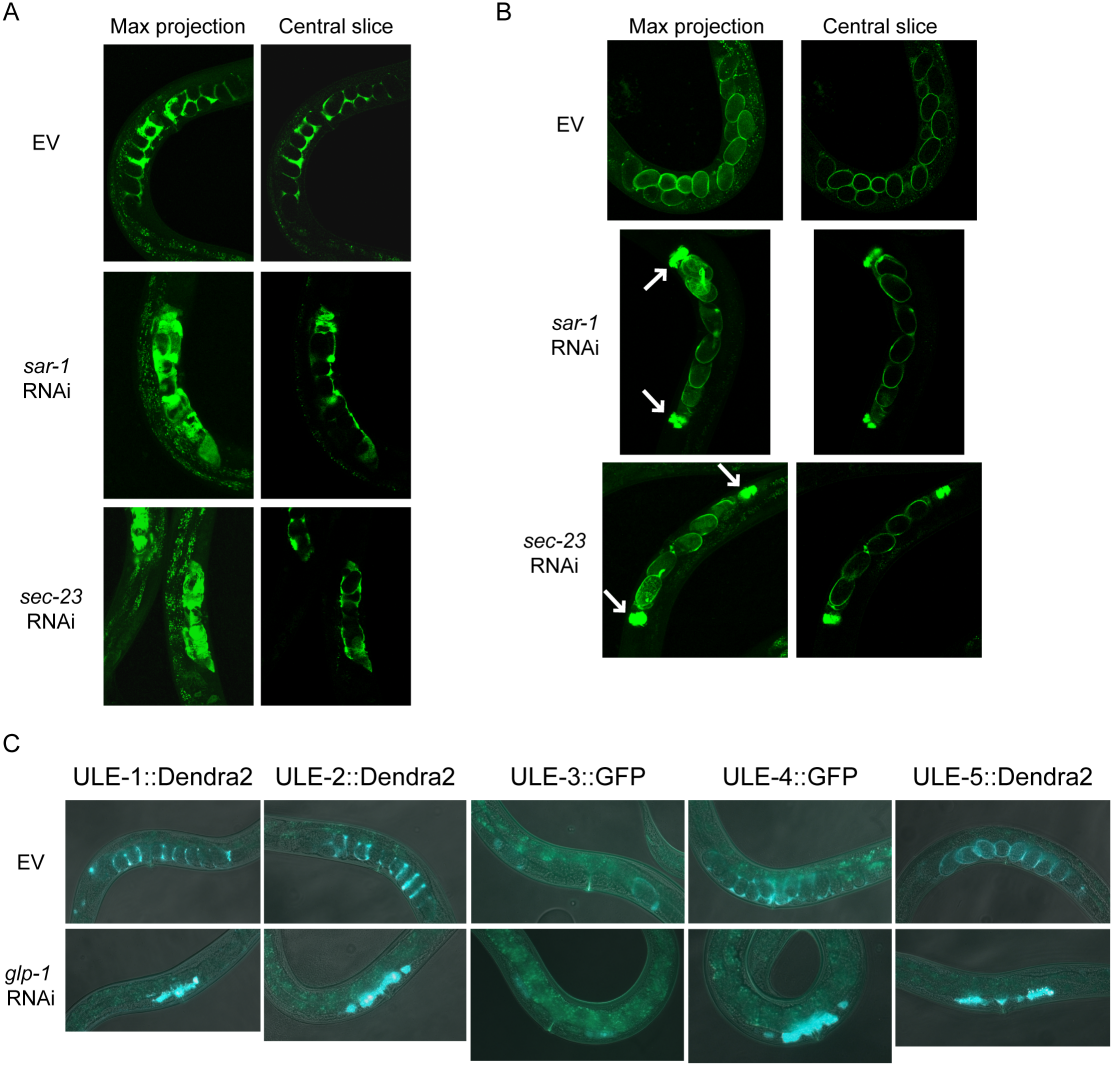
Uterine proteins are secreted by the somatic gonad. A-B. Uterine proteins are produced by the cells of the uterus (A) or spermatheca (B). Worms expressing uterine protein reporters were placed on empty vector control RNAi or RNAi against *sar-1* or *sec-23* at L4 and imaged 24 hours later. Representative images of worms expressing ULE-2::Dendra2 (A) and ULE-5::Dendra2 (B) are shown as a maximal projection of all images in the z-stack (left) or the central image of the stack (right). White arrows indicate the spermatheca. See S3 Fig for images of additional reporter lines. C. The presence of germ cells or embryos is not required for the expression of uterine protein reporters. Representative composite fluorescent and DIC images of day 1 adult worms expressing uterine protein reporters that were treated with empty vector (top) or *glp-1* RNAi (bottom) starting as embryos.

To determine which cells produce these secreted uterine proteins, we blocked protein secretion using RNAi against two genes encoding components of the COPII secretory vesicle coat complex, *sar-1* and *sec-23*. RNAi against either *sar-1* and *sec-23* has been previously shown to prevent protein secretion and trap fluorescent reporters in the cells that normally secrete them [27]. After 24 hours of RNAi treatment, fluorescent protein was only visible in the uterine cells (toroidal cells surrounding the uterus) in the ULE-1, ULE-2, and ULE-4 reporter lines (Fig 4A and S5A Fig). For the ULE-5 reporter line, fluorescence was predominantly visible in the spermatheca adjacent to the uterus (Fig 4B). We were not able to identify the cells secreting the fifth reporter (ULE-3), because GFP fluorescence was too dim to assess changes in expression pattern after *sar-1* and *sec-23* RNAi. We do not observe fluorescence in any other tissue of the worm in animals treated with *sar-1* and *sec-23* RNAi, suggesting that these uterine proteins are produced by the cells of the uterus or spermatheca and secreted locally into the uterine space.

### The germline is not required for the production of uterine proteins

To determine whether the presence of germ cells or fertilized eggs are required for uterine proteins to be expressed, we examined the effect of *glp-1* RNAi on our five fluorescent reporter strains. Loss of *glp-1* activity leads to sterility due to a failure of mitotic proliferation of the germline [28]. We observed that all five of these reporters were still expressed in the uterus of young adult *glp-1* RNAi treated animals, even in the absence of germ cells or developing embryos (Fig 4C). In addition, we asked whether sperm or oocytes specifically were required for uterine protein production by assessing uterine protein reporter expression in worms treated with *fog-2* and *mog-5* RNAi. *fog-2* is required for spermatogenesis in hermaphrodites and RNAi against *fog-2* leads to a feminization of the hermaphrodite germline [29, 30]. *mog-5* is required for the switch from spermatogenesis to oogenesis in the hermaphrodite germline, and loss of *mog-5* leads to masculinization of the germline [31]. Similar to our results with *glp-1* RNAi treated animals, we observed that all five uterine protein reporters were expressed in the uterus of young adult *fog-2* and *mog-5* RNAi treated animals (S5B Fig). Together, these results indicate the neither sperm, oocytes or the germline are required for expression of the ULE proteins.

Finally, we examined expression of the genes encoding the 17 adult-specific, age-increased proteins in *glp-4(bn2)* animals (which have few to no germ cells) using a previously generated DNA microarray dataset [32]. We found that none of the transcripts encoding these 17 proteins have significantly different expression levels in *glp-4* worms compared to controls. This result shows that the presence of germ cells, fertilized eggs, or developing embryos is not necessary for expression of these 17 proteins. Together with the data on secretion mutants, these data suggest that uterine proteins are produced by the somatic cells of the adult worm and secreted into the uterine lumen. However, it remains possible that these proteins could be subsequently transported inside the embryo while it is in the uterus, particularly in the case of the ULE-5 reporter that remains associated with the embryo after it has been laid.

### Age-increased uterine proteins are rapidly removed in young, but not old, animals

Increased abundance of a protein in old age could be caused by a decreased rate of protein removal. To test whether the removal rate of uterine proteins changes with age, we used the ULE-1 and ULE-2 reporters tagged with the photoconvertible fluorescent protein Dendra2 to examine the dynamics of these proteins in young and old worms.

**Fig 5:**
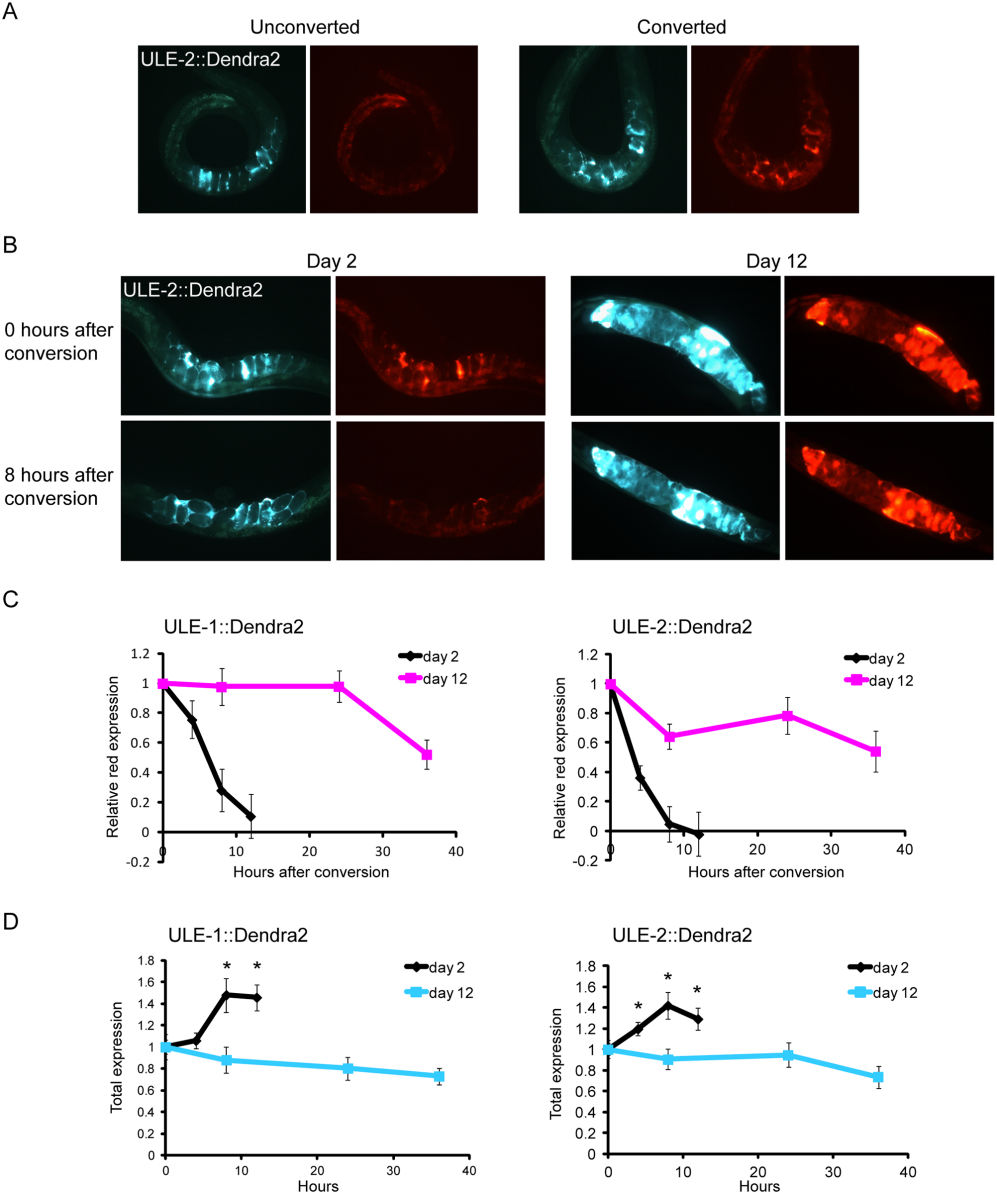
Uterine proteins turn over rapidly in young worms but not old. A. Representative images of day 2 adult worms expressing unconverted ULE-2::Dendra2 (left), or ULE-2::Dendra2 that has been photoconverted to the red form (right). B. Representative images of day 2 (left) and day 12 (right) adult worms expressing ULE-2::Dendra2 immediately after photoconversion (top), and re-imaged 8 hours later (bottom). C. Uterine proteins are rapidly removed in young worms but not in old. Dendra2 was photoconverted to the red form of the protein at time 0 in day 2 and day 12 adult worms expressing ULE-1::Dendra2 (left) and ULE-2::Dendra2 (right). The same worms were re-imaged at subsequent time points to monitor the decay of red protein expression. Shown are the levels of photoconverted red protein expression at each time point relative to red expression in the same worm at time 0 (n = 15 converted and 15 unconverted worms at day 2 and 13 converted and 15 unconverted worms at day 12 for the ULE-1 reporter line; n = 14 converted and 16 unconverted worms at day 2 and 11 converted and 11 unconverted worms at day 12 for the ULE-2 reporter line). Error bars are ± SEM. D. Total levels of ULE-1::Dendra2 (left) and ULE-2::Dendra2 (right) increase over 12 hours in day 2 adult worms. The data are represented as the average green fluorescent intensity of unconverted worms at each time point relative to that of the same worms at time 0 (n = ~10 worms in each condition). Error bars are ± SEM. *p<0.05 by Student’s t-test compared to expression at the same age at time 0.

Dendra2 is a monomeric fluorescent protein that can be irreversibly converted from a green to a red form by exposure to short wavelength light [33]. In unconverted worms, Dendra2 is green with essentially no background red fluorescence. After photoconversion, a population of red protein initially appears and declines with time as photoconverted protein is degraded or otherwise removed (Fig 5A). The level of red fluorescence can be monitored over time, allowing one to measure the rate of protein loss. Fusion proteins tagged with Dendra2 have been previously used to determine protein half-lives in tissue culture cells [34].

In order to interrogate the dynamics of Dendra2 reporters for ULE-1 and ULE-2, we photoconverted Dendra2 in day 2 and day 12 adults, re-imaged these photoconverted worms at several time points after conversion, and measured the decrease in red fluorescence intensity. We chose to examine day 2 adults as the young timepoint so that we could assess the effect of reproduction on uterine protein removal. We also imaged unconverted worms at the same time points to measure changes in total protein levels during the course of our experiment. Comparing the rate of protein loss to the change in total protein allows us to infer whether there is new protein production during the time span of the experiment.

Photoconverted red proteins were rapidly lost in young adult animals, with a half-life of less than 4 hours for ULE-2::Dendra2, and approximately 6 hours for ULE-1::Dendra2. However, in old animals, the half-life was greater than 36 hours for both Dendra2 reporters (Fig 5B and 5C). Two replicate experiments yielded nearly identical results regarding age-related changes in the dynamics of ULE-2::Dendra2 protein loss (S6 Fig). These results indicate that the removal rate of ULE-1::Dendra2 and ULE-2::Dendra2 protein is substantially slower in old animals than in young.

In young animals, the total levels of both ULE-1::Dendra2 and ULE-2::Dendra2 increased by approximately 50% over the course of the 12 hour experiment (Fig 5D). This increase in total protein level indicates that proteins are being rapidly synthesized in young worms. Together, these results show that ULE-1 and ULE-2 accumulate with age because protein production exceeds protein removal in young worms, and because protein removal rate substantially decreases in old worms.

### Prolonging reproduction delays the accumulation of age-increased uterine proteins

Why are uterine proteins removed slowly in old animals? One hypothesis is that the egg-laying process itself clears these proteins from the uterus in young worms. Since post-reproductive worms cannot lay fertilized eggs, they may fail to effectively remove uterine proteins. One of our five fluorescent reporters, ULE-5::Dendra2, is localized to the eggshell and is visible on eggs that have been laid (Fig 3B). We were unable to observe fluorescence outside the worm body after egg laying for the other four tagged uterine proteins. However, it is possible that they are not bound to the eggshell, so that even if they are excreted during egg-laying they may diffuse away too quickly to be observed.

If egg-laying or another aspect of progeny production is the major means of clearing age-increased uterine proteins, we hypothesized that extending the reproductive period of the animal might reduce their age-related accumulation. Self-fertile hermaphrodites lay eggs for the first 4 days of adulthood, at which time they are depleted of sperm. Mating hermaphrodites to males extends their reproductive period until day 8 of adulthood [35]. Therefore, mating to males allowed us to test whether continued reproduction could prevent the accumulation of uterine proteins in post-reproductive animals. We compared the expression of the five uterine protein reporters between day 5 self-fertile hermaphrodites (past the reproductive period) and day 5 mated hermaphrodites (producing progeny) to assess whether post-reproductive worms had higher levels of uterine protein than reproductive worms at the same age.

**Fig 6:**
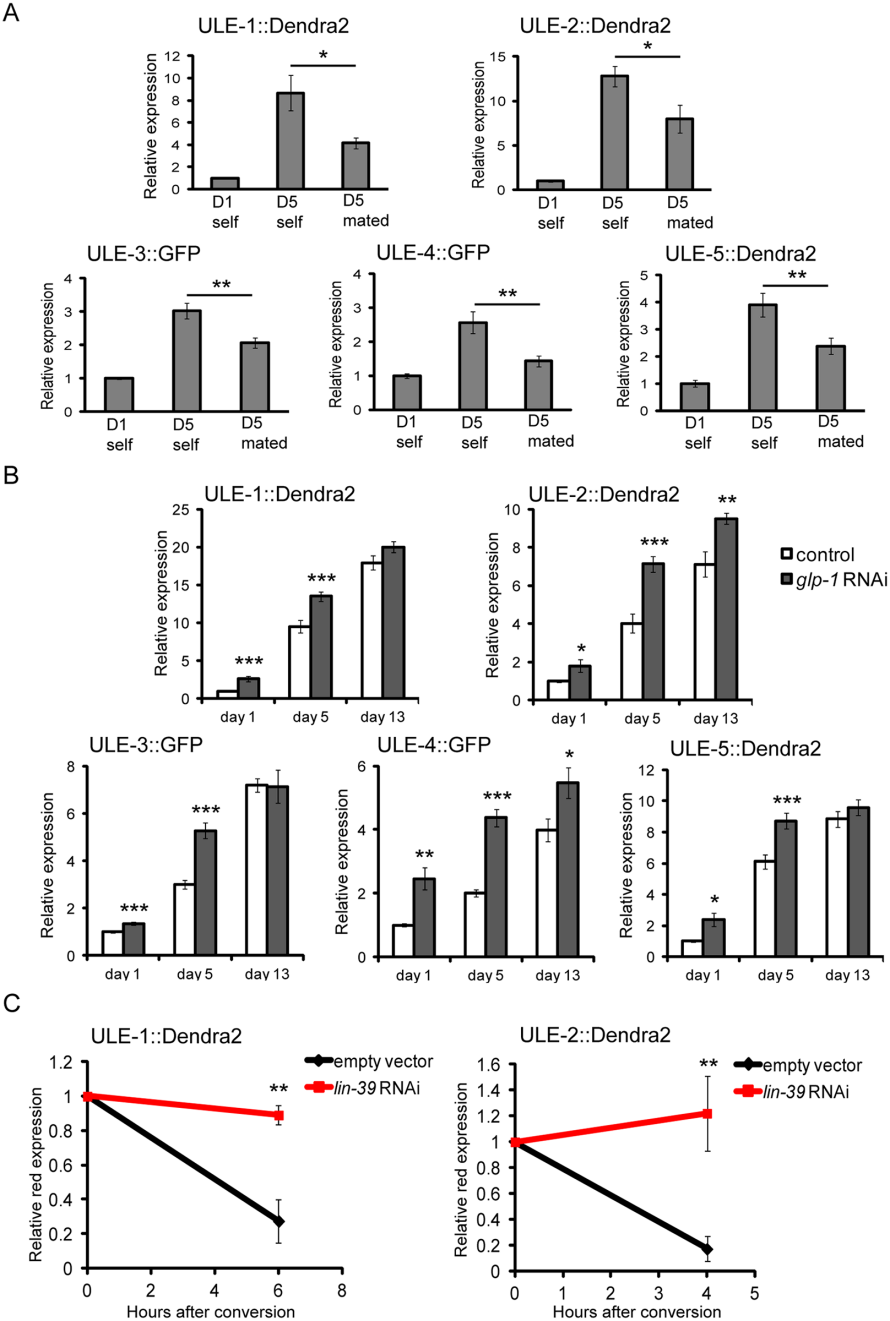
Uterine proteins are removed by egg-laying in young worms. A. Mating reduces the accumulation of all five uterine protein reporters. Day 1 adult hermaphrodite worms were placed with an equal number of male worms, allowed to mate for 48 hours, and imaged at day 5 of adulthood. Self-fertile worms were maintained without males and imaged at day 1 and day 5 of adulthood. The data are represented as mean fluorescent intensity relative to that of day 1 self-fertile animals (n = ~20 worms in each condition). Error bars are ± SEM. *p<0.05 and **p<0.01 by Student’s t-test, comparing day 5 self-fertile to day 5 mated animals. B. Uterine proteins accumulate more rapidly in *glp-1* RNAi treated animals. Worms were placed on empty vector control RNAi or RNAi against *glp-1* as L1 stage larvae and imaged on day 1, day 5, and day 13 of adulthood. The data are represented as mean fluorescent intensity relative to that of day 1 empty vector treated animals (n = ~20 worms in each condition). Error bars are ± SEM. *p<0.05, **p<0.01, and ***p<0.001 by Student’s t-test, comparing *glp-1* RNAi treated worms to empty vector treated worms at each timepoint. C. Vulvaless worms cannot remove uterine proteins. Worms expressing either ULE-1::Dendra2 (left) or ULE-2::Dendra2 (right) were placed on either empty vector control RNAi or RNAi against *lin-39* (which prevents vulva development) as L1 larvae. On day 1 of adulthood, Dendra2 was photoconverted and red protein expression was measured directly after conversion and 4 hours later (ULE-2::Dendra2) or 6 hours later (ULE-1::Dendra2). Shown are the levels of photoconverted red protein expression at 4 or 6 hours after conversion relative to red expression in the same worm at time 0 (n = ~10 converted and ~10 unconverted worms for each condition). Error bars are ± SEM. **p<0.01 by Student’s t-test comparing *lin-39* RNAi treated worms to empty vector controls at 4 or 6 hours. There is no significant difference between red expression at time 0 and 4 or 6 hours later for *lin-39* RNAi treated worms for either reporter (p>0.05 by Student’s t-test).

At day 5, mated hermaphrodites had approximately 50% the level of uterine protein reporter expression as unmated hermaphrodites for all five reporter lines (p<0.05 by Student’s t-test; Fig 6A). Fluorescent reporter expression increased 2.5 to 12-fold between day 1 and day 5 in unmated hermaphrodites, indicating that the increase in uterine protein abundance begins early in adult life. In mated animals, this increase in protein abundance was substantially blunted, though not completely abolished.

A second way to test whether the accumulation of uterine proteins can be delayed by extending reproduction is to use genetic perturbations that extend the reproductive period. RNAi of the genes *daf-2* and *moma-1* extends reproductive span, though to a lesser degree than mating [36]. RNAi treatment of *daf-2* but not *moma-1* also significantly extends lifespan [36]. We tested the effects of RNAi against these two genes on protein accumulation on the five uterine protein reporters. RNAi against *daf-2* significantly reduced the accumulation of all five uterine protein reporters at day 5 compared to control worms at day 5 (p<0.05 by Student’s t-test). RNAi against *moma-1* significantly reduced the levels of all the uterine protein reporters at day 5 except ULE-5::Dendra2, which had reduced expression but was not statistically significant (p = 0.09 by Student’s t-test; S7 Fig). Neither gene knockdown had an effect on uterine protein reporter expression at day 1. Together with the mating results, these results indicate that prolonging reproductive lifespan reduces uterine protein accumulation early in life.

Conversely, if egg-laying or another aspect of progeny production is responsible for removing uterine proteins in young worms, we would expect that worms that do not produce progeny would accumulate uterine proteins more quickly than wild-type animals. To test this, we compared the levels of our five uterine protein reporters in worms treated with empty vector or *glp-1* RNAi at day 1 (young reproductive), day 5 (young post-reproductive), and day 13 (old). We found that all five reporters had significantly higher levels of reporter expression in *glp-1* worms at day 1 and day 5, and two were also significantly higher at day 13 (p<0.05 by Student’s t-test; Fig 6B). These results indicate that uterine protein accumulation does occur more rapidly in worms that have never reproduced, and supports the hypothesis that reproduction is required for uterine protein removal in young worms.

Finally, to test more directly whether uterine proteins are removed by egg-laying, we asked whether the vulva is required for uterine protein removal in young worms. Eggs are normally laid through the vulva, and vulvaless mutant worms cannot lay any eggs. Therefore, if uterine proteins are removed by egg-laying in young worms, they should not be removed in young worms lacking a vulva. To test this possibility, we treated worms expressing either ULE-1::Dendra2 or ULE-2::Dendra2 with *lin-39* RNAi, which produces vulvaless worms [37]. We converted the tagged protein from green to red early on day 1 of adulthood (when most worms had only 1–2 fertilized eggs), and reimaged the worms 4 hours later for ULE-2 and 6 hours later for ULE-1 to measure the degree of protein removal. These timepoints were chosen because our previous data showed that at least 50% of red protein would be removed in wild-type worms. At these early timepoints, none of the *lin-39* RNAi treated worms had internal hatching of progeny. Any *lin-39* RNAi worm that was not vulvaless was discarded. 100% of control worms laid eggs in the 4 or 6 hours after photoconversion.

As in our previous data, we observed that approximately 75% of the red protein was removed in control worms in 4 hours for ULE-2::Dendra2 and 6 hours for ULE-1::Dendra2 expressing worms (Fig 6C). However, no significant amount of red protein was removed in worms treated with *lin-39* RNAi at the same time points. This suggests that in young worms, the predominant source of protein removal is by egg-laying (or another means of exit through the vulva). In summary, the results of the mating, reproductive mutants, *glp-1* mutants, and vulvaless mutants all indicate that uterine proteins are removed by egg-laying in young adults, and that the cessation of egg-laying contributes to the accumulation of uterine proteins with age.

### Simultaneous reduction of multiple age-increased proteins extends lifespan

Next, we asked whether the accumulation of proteins that increase in abundance with age is detrimental for lifespan. First, we reduced the expression of each of the 53 proteins that significantly changed abundance with age using RNAi starting at day 1 of adulthood and measured the resulting lifespan. We repeated this screen for the 17 age-increased, adult-specific proteins, this time starting the RNAi treatment at L1, so that the dsRNA against the gene would be present as soon as it became expressed. None of these single knockdowns showed a repeatable increase in lifespan (S4 Table).

However, we identified a group of adult-specific and secreted proteins, several of which are expressed in the uterus, that all increase in abundance with age. If the negative effect of protein accumulation is caused by this group as a whole, it may be necessary to reduce the levels of several proteins simultaneously in order to have an effect on lifespan. We chose to reduce the expression of several uterine proteins, as well as one other age-increased protein that is highly expressed (*far-6*). ULE-4, ULE-5 and FAR-6 have the highest levels of RNA [26] and protein (this study) in young adults (not including *vit-6*, which was excluded because vitellogenins are already known to have a detrimental effect on lifespan [38]). We attempted to express a GFP tagged version of *far-6* to determine if it was expressed in the uterine lumen, but we found that overexpression of this gene is toxic. ULE-2 and ULE-3 also relatively abundantly expressed, so that knockdown of these proteins might help alleviate overall accumulation of uterine proteins in the uterus.

We confirmed the effectiveness of the double, triple, and quadruple RNAi by examining the expression of the relevant uterine protein reporters after RNAi. Double, triple, and quadruple RNAi treatment substantially and significantly reduced the expression of the relevant reporters, although the RNAi knockdown effect in experiments involving two, three, and four simultaneous RNAi clones was reduced compared to the effect of single RNAi treatments (S8 Fig). We did not observe any defects in brood size, survival of embryos, or eggshell permeability in worms treated with *ule-4*, *ule-5*, and *far-6* RNAi (S9 Fig).

**Fig 7:**
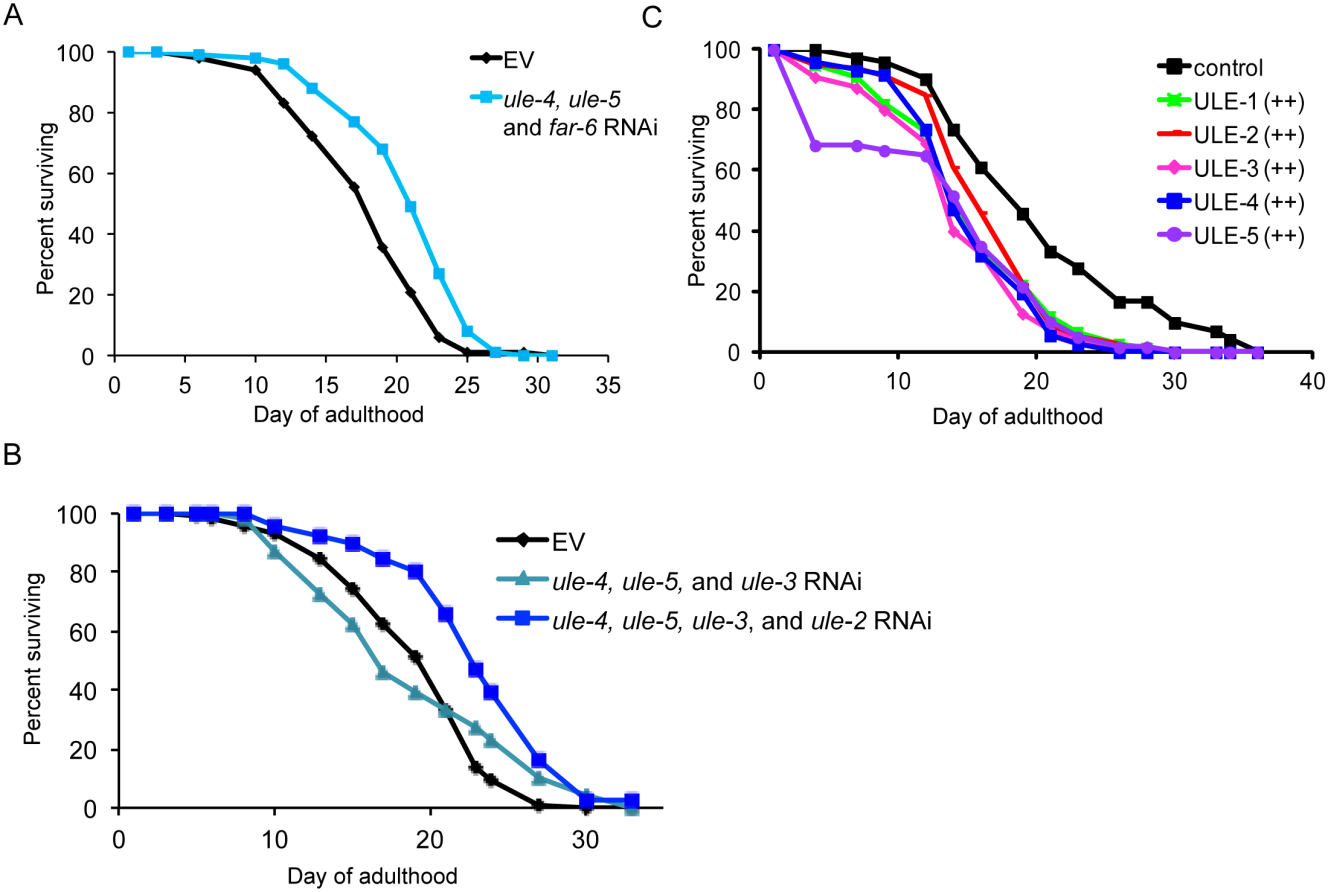
Simultaneously reducing the expression of multiple age-increased proteins extends lifespan, and overexpression of uterine proteins reduces lifespan. A. Knockdown of *ule-4*, *ule-*5, and *far-6* simultaneously by RNAi extends lifespan by approximately 20% (p = 5.3 x 10^−7^ by log rank test). Worms were placed on either empty vector control RNAi or an equal mixture of RNAi against *ule-4*, *ule-*5, and *far-6* as L1 stage larvae (n = 100 for each condition). Lifespan assay was performed three times (see S3 Table for additional lifespan data). Shown is a representative lifespan. B. Knockdown of *ule-4*, *ule-*5, and *ule-3* simultaneously by RNAi does not extend lifespan (p>0.05 by log rank test), while knockdown of *ule-4*, *ule-*5, *ule-3*, and *ule-2* simultaneously extends lifespan by approximately 20% (p = 3.5x10^-8^ by log rank test). Worms were placed on either empty vector control RNAi or an equal mixture of RNAi against the three or four uterine protein genes listed as L1 stage larvae (n = 100 for each condition). Lifespan assay was performed two times (see S3 Table for additional lifespan data). Shown is a representative lifespan. C. Overexpression of *ule-1*, *ule-2*, *ule-3*, *ule-4* or *ule-5* shortens lifespan by 20–30% (p<0.01 by log rank test for all five uterine proteins; n = 70–100 for each line). Overexpression lines contain each uterine protein tagged with either GFP or Dendra2 in extrachromosomal arrays in the *unc-119(ed3)* background. Transgenic lines expressing only the *unc-119* gene in an extrachromosomal array were used as controls. Two independent transgenic lines were tested for each *ule* gene. Shown are results from one representative line (see S3 Table for additional lifespan data).

We observed a 10–20% increase in median lifespan in worms treated with RNAi against *ule-4*, *ule-5*, and *far-6* in three biological replicates (p<0.01 by log rank test for all three replicates; Fig 7A and S4 Table). Reducing the expression of only *ule-4* and *ule-5* produced a 10–15% increase in lifespan in two out of three replicates (p<0.01 by log rank test; S4 Table). Reducing the expression of *ule-3*, *ule-4*, and *ule-5* simultaneously did not have a significant effect on lifespan in two biological replicates (Fig 7B and S4 Table). RNAi against *ule-3*, *ule-4*, *ule-5*, and *ule-2* simultaneously increased lifespan by 15–20% in two biological replicates (p<0.01 by log rank test; Fig 7B and S4 Table). These results suggest that the accumulation of individual uterine proteins is not detrimental for lifespan, but that the cumulative accumulation of multiple age-induced proteins or uterine proteins has a modest negative effect on lifespan.

Finally, to test whether increased expression of uterine proteins is sufficient to reduce lifespan, we generated overexpression lines for each of the five uterine proteins that carried extra copies of the protein tagged with either GFP or Dendra2 on an extrachromosomal array. We measured the lifespan of two independent overexpression lines for each of the five uterine proteins and observed a 20–30% reduction in lifespan in all lines compared to a transgenic control line (p<0.01 by log rank test; Fig 7C and S4 Table). This result is consistent with the possibility that accumulation of uterine proteins is detrimental for lifespan.

## Discussion

In this study, we identify a novel link between reproductive senescence and somatic aging in *C*. *elegans*. By assessing changes in protein abundance with age in an unbiased manner, we identify a previously uncharacterized class of proteins that accumulates with age in the adult uterus. The increase in uterine protein abundance in old animals is at least partially driven by age-induced infertility, which is a very early event in the aging process. Extending the reproductive period delays the accumulation of uterine proteins, and the presence of a vulva is required for the effective removal of uterine proteins in young animals. Therefore, the mid-life cessation of egg-laying likely contributes to the accumulation of uterine proteins with age. Finally, we show that simultaneous knockdown of multiple uterine proteins extends lifespan. These results suggest that the cessation of reproduction contributes to changes in the post-reproductive animal that are detrimental for survival.

Our findings provide a mechanistic link between aging of the reproductive system and aging of the soma. Many evolutionary theories of aging posit that the force of natural selection declines after the end of reproduction, because survival of the old parent provides little to no selective advantage for its offspring [39]. As a result, processes that are beneficial to the animals during development and reproduction may become dysregulated late in life [39, 40]. Uterine proteins are produced at high levels in young animals and are removed during the reproductive process. In old worms, the cessation of reproduction stops the removal of uterine proteins, leading to their accumulation with age. Though knockdown of single uterine proteins does not have an effect on lifespan, we show that knockdown of multiple uterine proteins simultaneously leads to a small but reproducible increase in lifespan. This suggests that high cumulative levels of uterine proteins is modestly detrimental for survival.

Despite this negative effect on the soma, it is likely that there is insufficient selective pressure in a post-reproductive animal to evolve a new outlet for these proteins and prevent their accumulation.

The accumulation of uterine proteins with age is conceptually similar to the post-reproductive accumulation of vitellogenins (yolk proteins). In young worms, these proteins are taken up by oocytes and are essential for viability of progeny. After reproduction ceases, the vitellogenins accumulate to toxic levels in the body cavity of the worm [3, 38]. In *C*. *elegans*, the age-related decline of the *elt-3* transcriptional circuit is another example of late life dysregulation of processes that are beneficial to the young animal. In young worms, *elt-3* is vital for function of the skin whereas in old worms, loss of *elt-3* function limits lifespan [7]. In mammals, the cell cycle regulator p16Ink4a increases expression with age in many human and mouse tissues and promotes cellular senescence [41, 42]. The regenerative capacity of many tissues declines with age, and reducing p16Ink4a expression can ameliorate these declines in many cell types [43–45].

A key difference between the uterine and yolk proteins and other examples of late life dysregulation of gene expression is that the timing of reproductive decline provides a mechanism for their accumulation. In many cases, the upstream causes of age-related changes in gene expression are not known, and therefore it is not clear what determines the kinetics of their age-related decline. For uterine and yolk proteins, the timing of their accumulation is tied to reproductive senescence. This provides a mechanistic link between the rate of aging of the germline and that of the soma and predicts that variation in the reproductive period (either experimentally or naturally occurring) could have a direct effect on somatic aging. Although the specific uterine proteins that we identify are not clearly conserved in mammals, the concept that the reproductive period has a direct role in specifying the rate of aging of the soma is likely to apply to other species as well.

What is the function of uterine proteins in young animals? *ule-1* was previously identified in mass RNAi screens to be required for normal progeny production [46, 47], and we confirmed in this study that knockdown of *ule-1* produces a small reduction in brood size. However, knockdown of *ule-4*, *ule-5*, and *far-6* simultaneously (which extends lifespan) had no effect on fertility or eggshell integrity. However, as these uterine proteins are expressed at very high levels in young animals and are actively produced over the reproductive period, it is likely that they do indeed have a function that remains to be discovered. It is possible that they are required redundantly for reproduction, and we did not achieve a sufficient degree of knockdown to observe a strong effect. It is also possible that they serve some non-reproductive function that we did not assay, or are beneficial only in particular environmental situations that are not recapitulated in the lab environment.

Many previous studies have examined potential trade-offs between reproductive capacity and somatic maintenance. One such study found that higher levels of progeny production in mated worms is a biomarker of aging and correlates with a longer lifespan [48]. This is consistent with the possibility that prolonged reproduction can be beneficial for lifespan. However, removing the entire germline extends lifespan, indicating that the presence of the germline generally accelerates aging of the soma [49–52]. It is unlikely that the longevity of germline-less mutants is mediated by uterine proteins, as we find that they are still expressed in animals lacking germ cells. Furthermore, removing the entire reproductive system (germ cells and somatic gonad, which includes the uterine cells and spermatheca that secrete uterine proteins) should eliminate uterine protein accumulation but does not increase lifespan [49]. Therefore, these observations indicate that the germline and somatic gonad have effects on lifespan independent of the effect of accumulation of uterine proteins.

Previous studies of uterine morphology during aging have observed a large increase in the size of the uterus in old worms [4, 53]. Much of this uterine hypertrophy can be attributed to the presence of uterine tumors, which are predominantly masses of DNA caused by the endoreduplication of unfertilized oocytes [4, 53, 54]. However, McGee et al (2012) also observed an increase in acellular material associated with the uterine masses, which is consistent with our observation that there is a general accumulation of extracellular protein in the uterus with age.

In addition to the five uterine proteins that we characterize in this study, we find that secreted proteins and proteins expressed specifically in adult animals generally increase in abundance with age. Several previous studies of the aging proteome in *C*. *elegans* also found that extracellular proteins increase in abundance with age, though the mechanism for this increase is unclear [19, 22]. A study that characterized newly synthesized proteins in young and old worms showed that extracellular proteins are enriched for having increased synthesis with age [22]. Therefore, some extracellular proteins might increase in abundance due to increased production, even as the protein synthesis rate declines with age generally [19, 20, 55].

Furthermore, it is likely that many of the additional secreted and adult-specific proteins that increase in abundance with age are also expressed in the uterus and increase by the same mechanism as the five that we examined in detail. We created fluorescent reporter proteins for five of 17 significantly age-increased, secreted, and adult-specific proteins, and found that all five are expressed in the uterine lumen. This suggests that there is a large class of extracellular uterine proteins in *C*. *elegans* that remain to be identified.

In summary, we found that uterine proteins accumulate in post-reproductive animals in part because of reproductive senescence itself. Therefore, the accumulation of uterine proteins is an example of an aging process that is at least partially driven by an intrinsic property of the animal (the length of the reproductive period), rather than extrinsic processes such as molecular damage. In addition, the post-reproductive accumulation of uterine proteins is a mechanistic example of how the timing of reproductive decline can in part determine the rate of somatic aging. It is likely that the duration of the reproductive period, a trait under strong selection, is an important determinant of lifespan in *C*. *elegans* as well as other species.

## Methods

### Preparation of protein lysates

For the proteomic analysis of aging, worms were grown on NGM plates supplemented with 30 mM 5-fluoro-2’-deoxyuridine (FUDR) to inhibit progeny production. During the reproductive period, worms were strained through a 40 μm pore nylon mesh each day to remove any contaminating eggs and larvae and transferred to new plates. Dead worms were manually removed from day 13 plates by picking before the worms were harvested for protein extraction. On day 4 and day 13 of adulthood, we collected approximately 10,000 worms into an equal volume of modified RIPA buffer (10 mM sodium pyrophosphate, pH 7.4, 150 mM NaCl, 1% Triton X-100, 1% sodium deoxycholate, 0.1% SDS, 2 mM EDTA) with protease inhibitors (Roche Complete, EDTA-free, #04693159001). The resulting pellet was frozen in liquid nitrogen and thawed three times, and manually disrupted by mortar and pestle until no large fragments of worm remained. The resulting samples were sonicated 3 times for 10 seconds each time on medium amplitude using a Diagenode Biorupter (UCD-200). The lysates were centrifuged at 2500xg for 30 minutes at 4°C to remove insoluble material and cell debris. We performed 3 biological replicates of this aging timecourse.

### Peptide labeling by reductive dimethylation

Protein lysates from three pairs of young and old biological replicates were precipitated by the addition of saturated trichloroacetic acid to 15% final concentration. The resulting protein pellet was washed five times with ice cold excess acetone. Proteins were resuspended in 8 M urea, 100 mM NaCl, 25 mM Tris, pH 8.2 in the presence of protease inhibitor cocktail (Roche Complete, EDTA-free, #04693159001). Proteins were reduced by the addition of dithiothreitol (DTT) to 5 mM final concentration and incubation at 50°C for 30 min. Cysteines were alkylated by the addition of iodoacetamide to 14 mM final concentration and incubation at room temperature for 1hr in the dark. The alkylation reaction was quenched by increasing the final concentration of DTT to 10 mM. After diluting the samples to 1 M urea, proteins were proteolytically digested with 5 ng/ml trypsin (Sequencing Grade Modified Trypsin, Promega, #V5111). Enzymatic digestion was quenched by the addition of trifluoroacetic acid to 0.1% final concentration, pH < 2. The resulting peptides were desalted using Sep-Pak C18 columns (Waters, # WAT023590). Peptides were dried down and resuspended in 1 M HEPES, pH 7.5. Peptides were chemically labeled by reductive dimethylation of lysine residues and N-termini as previously described [23]. Peptides derived from young worm lysates were light-labeled (+28Da) by reaction with 4% d0-formaldehyde and 600 mM sodium cyanoborohidride for 10 minutes at room temperature. Peptides derived from old worm lysates were heavy-labeled (+34Da) by reaction with 4% formaldehyde- d_2_ and 600 mM sodium cyanoborodeuteride for 10 minutes at room temperature. Each reaction was repeated and then quenched by the addition of 10% formic acid to pH 3. Quenching was aided by bath sonication for 1 hour. Light- and heavy-labeled pairs were mixed one-to-one and subsequently desalted using Sep-Pak C18 columns.

### Strong cation exchange chromatography

Light- and heavy-labeled peptide mixtures were then chromatographically fractionated using strong cation exchange chromatography on an Agilent 1200 high-performance liquid chromatography instrument (Agilent Technologies). Peptides were loaded on to a Polysulfoethyl A column (Poly LC Inc., #209SE502) in buffer A (7 mM KH_2_PO_4_, 30% Acetonitrile, pH 2.65) and eluted with a gradient of 0–25% buffer B (7 mM KH_2_PO_4_, 350 mM KCl, 30% Acetonitrile, pH 2.65) for 29 minutes, 25–100% buffer B for 5 minutes, and 100% buffer B for 5 minutes. Twelve fractions were collected and desalted using Sep-Pak C18 columns.

### Liquid chromatography-tandem mass spectrometry

Peptides from each fraction were analyzed on an LTQ Velos Orbitrap mass spectrometer (Thermo Fisher Scientific) coupled to an Agilent 1100 high performance liquid chromatography pump (Agilent Technologies) and a MicroAS autosampler (Thermo Scientific). Approximately 10% of each fraction was loaded on to a 17cm fused silica microcapillary column (100um inner diameter) with an in-house pulled tip (~5 um inner diameter) packed with C18 reversed-phase resin (Magic C18AQ, Michrom Bioresources). Peptides were eluted into the mass spectrometer’s nanospray ionization source via a two-step gradient of 7–25% buffer B (2.5% water and 0.1% formic acid in acetonitrile (v/v)) in buffer A (2.5% acetonitrile and 0.1% formic acid in water (v/v)) over 60 minutes followed by a second phase of 25–45% buffer B over 20 minutes. The mass spectrometer collected 10 ion-trap MS/MS spectra per data-dependent cycle.

### Mass spectrometry data analysis

Raw data acquired by the mass spectrometer were converted to the mzXML format, and MS and MS/MS data were extracted using in-house software ([56, 57]; software available by request). Spectra were analyzed using the Sequest (version 27, revision 12) algorithm and the Uniprot *C*. *elegans* protein sequence database using the target-decoy strategy [58]. Search parameters included tryptic cleavage, two missed cleavages allowed, peptide mass tolerance of 50 ppm, static carboxyamidomethylation of cysteine residues (+57.02146 Da), differential oxidation of methionine residues (+15.99491 Da), static demethylation of lysine residues and N-termini (+28.03230 Da), and differential modification of lysine residues and N-termini (+6.037660 Da). Peptide-spectrum matches were determined with an estimated false discovery rate <1% and quantification of light- and heavy-labeled peptides was performed by the Vista quantitative analysis tool [56]. The mass spectrometry proteomics data have been deposited to the ProteomeXchange Consortium via the PRIDE partner repository with the dataset identifier PXD002432.

### Analysis of the aging proteome

The resulting heavy/light ratios for each protein were log_2_ transformed and median centered to control for inexact mixing. Therefore, proteins that changed in this experiment are those that changed relative to all proteins, rather than on an absolute per worm basis. Only proteins that were identified in at least two of the three replicates were considered for further analysis (1796 proteins). Proteins that significantly changed across replicates were identified using the rank product algorithm as previously described [24]. The false discovery rate corresponding to each rank product was calculated by 10,000 random permutations of the ranks for each of the three replicates. At a 10% false discovery rate, 53 proteins significantly changed in abundance with age.

Gene set enrichment analysis was performed as previously described [59], using *C*. *elegans* gene ontology terms as gene sets and including annotations inferred electronically. False discovery rates were calculated using 1000 permutations of the data. Only those gene ontology terms that were enriched with an FDR<0.05 are reported as significant.

To analyze the developmental expression pattern of each protein, we used stage-specific RNA-seq data generated by the ModEncode consortium [26]. RPKM values were row normalized to obtain the relative expression of each gene in each stage (independent of the absolute level of expression of that gene). Hierarchical clustering of the developmental expression pattern of all proteins was performed in MATLAB (MathWorks Incorporated, R2010b) with the clustergram function using the “correlation” distance metric.

### Strains

All *C*. *elegans* strains were handled and maintained as described previously [60].

Worms were maintained and all experiments were performed at 20°C. The N2 strain was used for all experiments unless otherwise stated.

GFP and Dendra2 reporter constructs and overexpression lines were generated by cloning the complete coding sequence and introns, 5’ and 3’ UTRs, and 600–1000 base pairs of upstream and 200–1000 base pairs of downstream flanking sequence (generally to start or end of the adjacent gene) of each protein of interest into the pCFJ350 or pCFJ352 vector [61]. These vectors also contain the *C*. *briggsae unc-119* gene as a transgenic marker. We inserted either eGFP or Dendra2 into the C-terminus of the protein directly upstream of the stop codon by Gibson assembly [62]. Transgenic worms were produced by microinjection of the resulting plasmid into the *unc-119(ed3)* background to form extrachromosomal arrays. A list of all of the strains used in this study and the primers used to clone each gene can be found in S4 Table.

### RNAi

All RNAi experiments were carried out on NGM plates supplemented with 100 ug/mL ampicillin, and 1 mM IPTG (2 mM IPTG if using FUDR). Plates were seeded with 10x concentrated overnight cultures of *E*. *coli* expressing the appropriate RNAi clone or control. Most RNAi clones were obtained from the Ahringer RNAi library [63] and sequenced to verify proper insertions. HT115(de3) *E*. *coli* carrying the L4440 empty vector plasmid were used as a control for all experiments.

For some proteins of interest, the corresponding RNAi clone was not present in the Ahringer RNAi library, did not grow, or was incorrect when sequenced. For these proteins, we constructed RNAi vectors by cloning a 500–1500 base pair fragment of the gene into the L4440 vector at the EcoRV restriction site. The resulting plasmids were transformed into the HT115(de3) strain of *E*. *coli*. A list of these strains and the primers used to clone the inserts can be found in S4 Table.

When multiple RNAi clones were used simultaneously in an experiment, the cultures were mixed such that the final concentration of *E*. *coli* was the same as the single RNAi clone and control conditions.

### Imaging and quantification of expression

Synchronized worms at the relevant age were immobilized on slides with 1 mM levamisole and imaged on a Zeiss Axioplan fluorescent microscope. All conditions that are quantitatively compared were imaged on the same day using the same microscope and image capture settings. Representative images of conditions that are not quantitatively compared may have been taken using different settings in order to best display the expression pattern. The average pixel intensity in the uterus of each worm was quantified using ImageJ [64] and background subtracted using the average pixel intensity of at least two representative background areas outside the worm. Regions of interest were selected manually.

### Secretion experiment

Late L4 worms of the relevant reporter strain were placed on plates seeded with *sar-1*, *sec-23*, or empty vector RNAi. Worms were imaged 24 hours later on a Leica TCS SP8 confocal microscope using the 20x objective at 1024x1024 pixels. Images were obtained as Z stacks at 1.04 μm intervals. The top and bottom of the worm (first and last image in the stack) were selected manually. Images are displayed as either as a maximum projection of all the images in the Z-stack, or just the center image in the Z-stack.

### Mating experiment

For the mating experiment, synchronized adult worms of the relevant reporter strain were either maintained as self-fertile hermaphrodites, or placed with an equal number of N2 males on day 1 of adulthood. Total worm number was kept the same on self-fertile and mating plates (for example, 50 hermaphrodites on the self plates and 25 hermaphrodites and 25 males on the mating plates). Males were removed two days later on day 3 of adulthood, and both mated and self-fertile hermaphrodites were transferred to fresh plates. Mating success was judged by the presence of male progeny on the plate and the continued production of fertilized eggs at day 5. Self-fertile worms were imaged at day 1 and day 5 of adulthood, and mated worms at day 5.

### Dendra2 photoconversion and measurement of protein dynamics

For the Dendra2 conversion experiments, worms were mounted on 7% agarose pads using 0.2% tricaine and 0.02% levamisole in M9 as anesthetic. Coverslips were sealed to the slide with petroleum jelly. Worms were mounted on individual slides and recovered to individual plates after imaging so that the data from each worm could be analyzed separately.

Dendra2 was converted by scanning single worms with a 405 nm 50 mW diode laser at 100% output for 60 s using a 20x objective at 1024x1024 pixels. Converted and unconverted worms were imaged on a Zeiss Axioplan microscope using the GFP filter to visualize the green form of the protein and the TRITC filter to visualize the red form. Worms were imaged immediately after conversion and then recovered to individual seeded NGM plates at 20°C. At the indicated timepoints after conversion, individual worms were re-mounted on slides and re-imaged following the same procedure as above, and then returned to seeded plates. Microscope and imaging settings were kept constant between imaging timepoints. Both converted worms and unconverted worms that had otherwise been treated identically were imaged at each timepoint. Any worm that died during the course of the experiment was not considered in the final analysis.

The red expression values for each worm were background subtracted using the average red expression in unconverted worms as background. For each worm at each timepoint, the background subtracted red expression value was normalized to that same worm’s expression right after conversion (time 0). Therefore, a relative expression value of 1 means that the worm’s expression is the same as at time 0, while a relative expression value of 0 means that the worm’s expression is equal to the background red expression level in unconverted worms.

Absolute levels of red or green protein are not directly comparable between young and old worms, as the images were taken using different settings to best capture the full range of pixel intensities between converted and unconverted worms. Therefore, all comparisons are relative to expression at time 0 for each condition.

### Lifespan experiments

Lifespan assays were performed as previously described [65]. All lifespan assays were carried out at 20°C. Worms were maintained on NGM plates supplemented with 30 mM FUDR to inhibit progeny production. In experiments using RNAi, 100 ug/mL ampicillin and 2 mM IPTG were used to select for and induce RNAi bacteria. Animals that died due to internal progeny hatching or bursting were censored. Animals were scored as dead if they failed to respond to prodding by a pick. Significance of lifespan experiments was determined using the log-rank test [66].

### Fertility experiments

Synchronized L1 worms were placed on NGM plates seeded with the appropriate RNAi clone or empty vector control. At the L4 stage, individual unmated hermaphrodites were placed on a new RNAi or control plate and transferred every 24 hours until day 4 of adulthood. The number of eggs laid by each worm was counted each day. 48 hours later, the percent of eggs that had hatched and developed to L4 larvae stage was counted.

### Eggshell permeability experiment

Mixed stage embryos that had been laid by day 1 adult worms raised on the indicated RNAi clone since L1 were stained with 20 ng/mL 4’,6-diamidino-2-phenylindole (DAPI) in an isotonic buffer (150 mM KCl, 5 mM HEPES, pH 7.5), as previously described [67].

## Supporting Information

S1 Fig Comparisons to previous studies of the aging proteome. A. Proteins that change in abundance in a previous study of the aging proteome in *C*. *elegans* [22] generally change in the concordant direction in this study. The data are displayed as heat maps showing average log_2_ ratios of (old/young) expression of proteins that significantly increased or decreased in abundance at day 5 compared to day 1 in the Liang et al study (VL) that were also present in our study (SMZ). 70% of proteins that increased in abundance in the Liang et al study also increased in our study (p<10^−5^ by binomial test), and 76% of the proteins that decreased in abundance in the Liang et al study also decreased in our study (p<10^−15^ by binomial test). However, the aging fold changes of proteins in the two studies were only modestly correlated (R^2^ = 0.26). B. Proteins that change in abundance in a previous study of the aging proteome in *C*. *elegans* [19] generally change in the concordant direction in this study. The data are displayed as heat maps showing average log_2_ ratios of (old/young) expression of proteins that increased or decreased in abundance at least 1.5-fold between day 1 and day 12 in the Walther et al study (DMW) that were also present in our study (SMZ). 72% of proteins that increased in abundance in the Walther et al study also increased in our study (p<10^−12^ by binomial test), and 84% of the proteins that decreased in abundance in the Walther et al study also decreased in our study (p<10^−8^ by binomial test). However, the aging fold changes of proteins in the two studies were only modestly correlated (R^2^ = 0.33). C. Proteins that were previously found to be age-insoluble [17, 18] do not generally change in abundance in this study. The bar graphs show the fraction of proteins that were determined to be age-insoluble in two previous studies for all 1796 proteins covered in this study (all), the proteins that significantly increased in abundance (up), or proteins that significantly decreased (down). There was no significant enrichment for age-insoluble proteins in either the proteins that increased or decreased in abundance in this study (p>0.05 by Fisher’s exact test). D. The histograms show the distribution of aging fold changes of all 1796 proteins covered in our data (black) and the 601 (left) or 185 proteins (right) that were determined to be age-insoluble in previous studies (blue). Insoluble proteins are not generally shifted towards being increased or decreased in abundance with age.
(TIF)

S2 Fig Secreted and adult-specific proteins increase in abundance with age. A. Secreted proteins tend to increase in abundance with age. The histogram shows the distribution of aging fold changes of all 1796 proteins covered in our data (black), the 249 proteins that are predicted to be secreted (yellow) and were covered in our data, and the same set of secreted proteins minus the 34 proteins that also significantly increase in abundance with age (orange). Secreted proteins are significantly shifted towards increasing in abundance with age (p < 10^−27^ by Kolmogorov-Smirnov test), even when the 34 proteins that are both significantly increased and secreted are removed (p < 10^−17^ by Kolmogorov-Smirnov test). B. Adult-specific proteins tend to increase in abundance with age. The histogram shows the distribution of aging fold changes of all 1796 proteins covered in our data (black), the 95 proteins whose transcripts are expressed specifically in adults (light blue), and the same set of adult-specific proteins minus the 17 proteins that also significantly increase in abundance with age (dark blue). Adult-specific proteins are significantly shifted towards increasing in abundance (p < 10^−10^ by Kolmogorov-Smirnov test), even when the 17 proteins that are both significantly increased in abundance with age and adult-specific are removed (p < 10^−4^ by Kolmogorov-Smirnov test). C. The Venn diagram (left) shows the overlap between the 95 proteins whose transcripts are expressed specifically in adults and the 249 proteins that are predicted to be secreted. 55 proteins are both adult-specific and secreted (a 4-fold enrichment over expectation, p<10^−24^ by Fisher’s exact test). The histogram (right) shows the distribution of aging fold changes of these 55 proteins (green) compared to all 1796 proteins covered in our experiment (black). The proteins that are both secreted and expressed specifically in adults are strongly shifted towards increasing in abundance with age (p < 10^−18^ by Kolmogorov-Smirnov test). D. Proteins that increase in abundance with age have similar absolute expression levels to all proteins in adults, but lower absolute expression levels in earlier developmental stages. The data are represented as mean read count (RPKM) in each stage for all proteins (black) and for the 40 proteins that significantly increase in abundance with age (red). Error bars are ± SEM. Developmental RNA-seq data was generated by ModEncode [26]. EE = early embryo; LE = late embryo; L1–4 = larval stage 1–4; YA = young adult.
(TIF)

S3 Fig Expression of uterine proteins in larvae. Representative images of the five uterine protein reporters in early larvae (L2/L3), L4 larvae, and young adult males (day 1–2 of adulthood). White arrows indicate fluorescent protein expression. “No expression” means that no fluorescent protein expression was visible at 63x magnification. Proteins were tagged at the C-terminus with eGFP (ULE-3, ULE-4) or Dendra2 (ULE-1, ULE-2, ULE-5). At least 20 worms of each reporter at each stage were examined.
(TIF)

S4 Fig Effect of FUDR and RNAi against uterine proteins on uterine protein reporter expression. A. Autofluorescence increases 30% between day 4 and day 13 in non-transgenic N2 control animals. The data are represented as mean fluorescent intensity at day 13 relative to mean fluorescent intensity at day 4 (n = ~20 worms in each condition). Error bars are ± SEM. B. RNAi against each ULE gene reduces expression of its reporter at least 3-fold at both day 4 and day 13 for all five lines (p<0.001 by Student’s t-test for RNAi vs. control treated worms for all lines at both timepoints). Worms expressing uterine protein reporters were placed on empty vector control or the ULE gene RNAi as L1 larvae and imaged as day 4 and day 13 adults (n = ~20 worms for each line at each timepoint). The data are shown as mean fluorescent intensity minus the mean expression in a non-transgenic control worms fed empty vector RNAi. Error bars are ± SEM. C. Effect of FUDR on uterine protein reporter expression. Worms expressing uterine protein reporters were grown in the presence or absence of 30 mM FUDR starting at day 1 of adulthood and imaged at day 4 and day 13. Expression of ULE-2::Dendra2 increased 70% at day 4 in worms grown without FUDR, while ULE-5::Dendra2 expression decreased 20% at day 13 in worms grown without FUDR. The data are represented as mean fluorescent intensity relative to that of day 4 FUDR treated animals (n = ~20 worms in each condition). Error bars are ± SEM. *p<0.05 and **p<0.01 by Student’s t-test, comparing FUDR to no FUDR at each timepoint.
(TIF)

S5 Fig Expression of uterine proteins in secretion mutants, masculinized, and feminized worms. A. Uterine proteins are produced by the uterine cells (shown in Fig 4A and this figure) or the cells of the spermatheca (shown in Fig 4A). Worms expressing uterine protein reporters were placed on empty vector control RNAi or RNAi against *sar-1* or *sec-23* at L4 and imaged 24 hours later. Representative images of worms expressing ULE-1::Dendra2 (left) or ULE-4::GFP (right) are shown as a maximal projection of all images in the z-stack or the central image of the stack. B. Oocytes and sperm are not required for uterine protein reporter expression. Representative fluorescent (top) and DIC (bottom) images of day 1 adult worms expressing uterine protein reporters that were treated with empty vector, *mog-5*, or *fog-2* RNAi starting as embryos.
(TIF)

S6 Fig Consistent measurement of protein dynamics in a replicate experiment. Biological replicate experiment of the measurement of protein dynamics of ULE-2::Dendra2 shown in Fig 5. Left: Dendra2 was photoconverted to the red form of the protein at time 0 in day 2 and day 12 adult worms expressing ULE-2:Dendra2. The same worms were re-imaged at subsequent time points to monitor the decay of red protein expression. The red expression values for each worm were background subtracted using the average red expression in unconverted worms as background. The data are represented as the average background subtracted red expression at each time point relative to the background subtracted red expression of the same worm at time 0 (n = 12 converted and 17 unconverted worms at day 2, and 16 converted and 16 unconverted worms at day 12). Right: The average green fluorescent intensity of unconverted worms at each time point relative to that of the same worms at time 0 (n = X). Error bars are ± SEM.
(TIF)

S7 Fig Mutants that extend reproductive span blunt the accumulation of uterine protein reporters. Worms expressing uterine protein reporters were placed on empty vector control RNAi or RNAi against *daf-2* or *moma-1* as L1 stage larvae and imaged on day 1 or day 5 of adulthood. The data are represented as mean fluorescent intensity relative to that of day 1 empty vector control treated animals (n = ~20 worms in each condition). Error bars are ± SEM. *p<0.05, **p<0.01, and ***p<0.001 by Student’s t-test, comparing day 5 empty vector control worms to day 5 *daf-2* or *moma-1* RNAi worms. There is no significant effect of *daf-2* or *moma-1* RNAi on the expression of any of the five reporters at day 1.
(TIF)

S8 Fig RNAi controls. A. RNAi knockdown of *ule-4* and *ule-5* by mixed RNAi treatment is effective. Wild-type worms and worms expressing ULE-4::GFP or ULE-5::Dendra2 were placed on empty vector control RNAi, either *ule-4* RNAi or *ule-5* RNAi (single RNAi), an equal mixture of *ule-4* RNAi and *ule-5* RNAi, or an equal mixture of *ule-4*, *ule-*5, and *far-6* RNAi as L1 larvae and imaged as day 3 adults. Single RNAi treatment reduced the level of both reporters to that of non-transgenic worms. Double and triple RNAi treatments substantially reduced the expression of both reporters but had slightly reduced effectiveness (*p<0.05 by Student’s t-test comparing RNAi treated worms to a non-transgenic control). The data are shown as mean fluorescent intensity minus the mean expression in a non-transgenic control worms fed empty vector RNAi. Error bars are ± SEM. B. RNAi knockdown of *ule-4*, *ule-5*, *ule-3*, and *ule-2* by mixed RNAi treatment is effective. Wild-type worms and worms expressing uterine protein reporters were placed on empty vector control RNAi, RNAi against just the reporter gene (single RNAi), an equal mixture of *ule-4* RNAi, *ule-5* RNAi, and *ule-3* RNAi, or an equal mixture of *ule-4* RNAi, *ule-5* RNAi, *ule-3* RNAi, and *ule-2* RNAi as L1 larvae and imaged as day 3 adults. Single RNAi treatment reduced the level of all reporters to that of non-transgenic worms. Triple and quadruple RNAi treatments reduced the expression of all reporters but had reduced effectiveness (*p<0.05 by Student’s t-test comparing RNAi treated worms to a non-transgenic control). The data are shown as mean fluorescent intensity minus the mean expression in a non-transgenic control worms fed empty vector RNAi. Error bars are ± SEM.
(TIF)

S9 Fig Fertility phenotypes after uterine protein knockdown. A. Knockdown of *ule-4*, *ule-*5, and *far-6* simultaneously does not affect the number of eggs laid, but knockdown of *ule-1* produces a modest reduction in brood size. Worms in the first larval stage were placed on either empty vector control RNAi, an equal mixture of *ule-4*, *ule-*5, and *far-6* triple RNAi, or *ule-1* single RNAi and the number of eggs laid on days 1–4 of adulthood were counted. Data is shown as the mean total number of eggs laid per worm in the first four days of adulthood (n = ~10 worms per condition). Error bars are ± SEM. *p<0.05 by Student’s t-test compared to empty vector control worms. B. Single knockdown of *ule-1* or triple knockdown of *ule-4*, *ule-*5, and *far-6* does not affect survival of progeny. Worms in the first larval stage were placed on either empty vector control RNAi, an equal mixture of *ule-4*, *ule-*5, and *far-6* triple RNAi, or *ule-1* single RNAi and the number of eggs laid on days 1–4 of adulthood were counted. The percent of eggs that had hatched and developed at least to the fourth larval stage were counted 48 hours later. Data is shown as the mean proportion of eggs surviving that were laid on each of the first four days of adulthood (n = ~10 worms per condition). Error bars are ± SEM. C. Knockdown of *ule-4*, *ule-*5, and *far-6* simultaneously by RNAi (triple RNAi) does not increase permeability of the eggshell to DAPI. RNAi against *perm-1* was used as a positive control [68]. N2 worms were placed on the appropriate RNAi clone as L1 stage larvae and mixed stage embryos from the resulting day 1 adults were stained with 20 ng/mL DAPI. Representative composite fluorescent and DIC images (left) show DAPI positive *perm-1* treated embryos and DAPI negative triple RNAi treated embryos. The graph (right) shows the percentage of embryos that were DAPI positive (n = 5 slides per condition, > 50 embryos per slide). Error bars are ± SEM.
(TIF)

S1 Table 1796 proteins identified in at least two of three biological replicates. (CSV)

S2 Table 53 proteins that significantly changed with age by rank-product analysis. Proteins are ordered as displayed in the heatmap in Fig 1A.
(CSV)

S3 Table Gene ontology terms that are enriched for changing abundance with age by gene set enrichment analysis. (XLSX)

S4 Table Additional lifespan data. (XLSX)

S5 Table Strains used in this study. (XLSX)
