# Supplementary figures and images for: Reproductive Aging Drives Protein Accumulation in the Uterus and Limits Lifespan in C. elegans
Source: PLoS Genet. 2015 Dec 11;11(12):e1005725. doi: 10.1371/journal.pgen.1005725 (PMC4676719; doi:10.1371/journal.pgen.1005725)

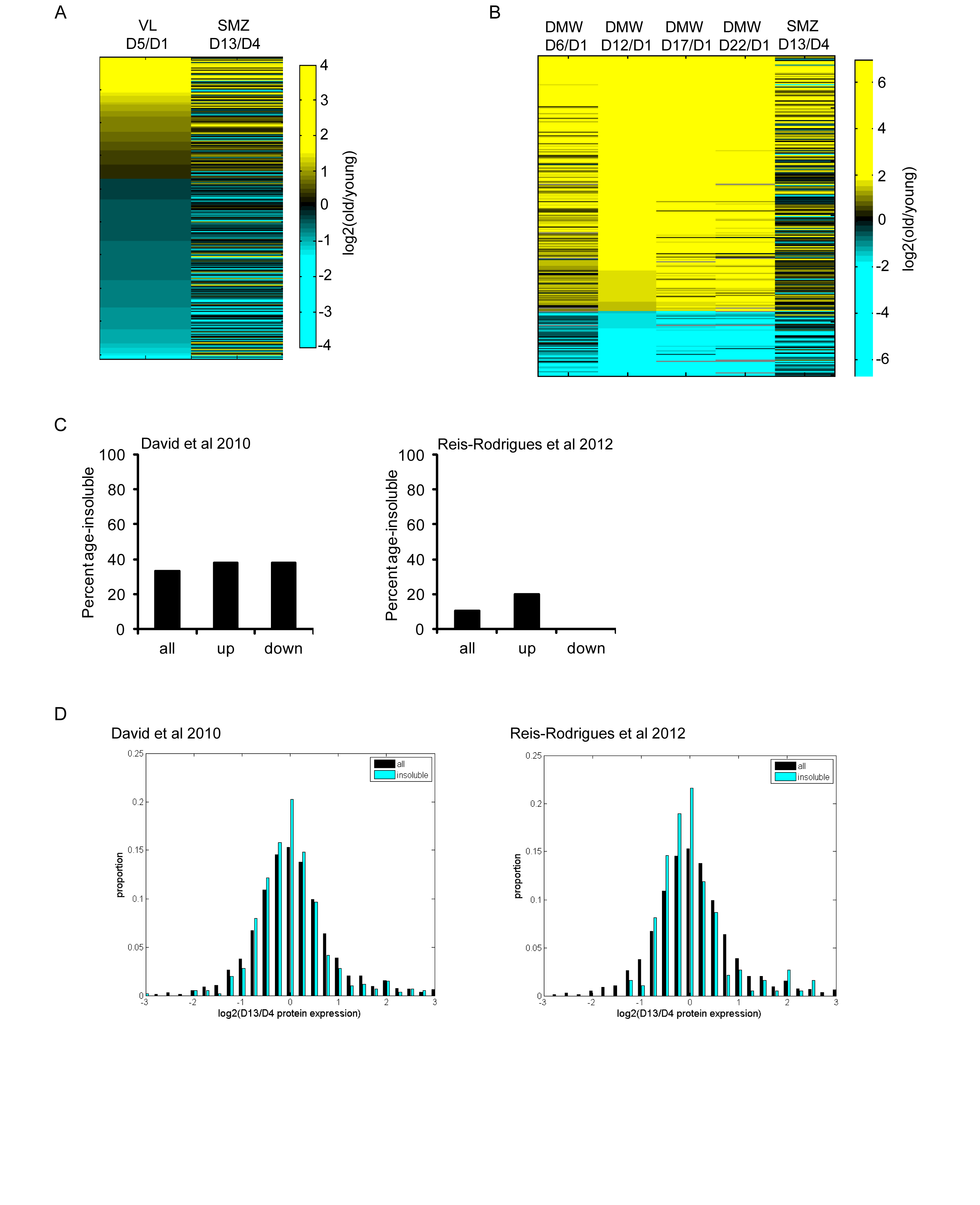

Supplement: S1 Fig — A. Proteins that change in abundance in a previous study of the aging proteome in C. elegans [22] generally change in the concordant direction in this study. The data are displayed as heat maps showing average log2 ratios of (old/young) expression of proteins that significantly increased or decreased in abundance at day 5 compared to day 1 in the Liang et al study (VL) that were also present in our study (SMZ). 70% of proteins that increased in abundance in the Liang et al study also increased in our study (p<10−5 by binomial test), and 76% of the proteins that decreased in abundance in the Liang et al study also decreased in our study (p<10−15 by binomial test). However, the aging fold changes of proteins in the two studies were only modestly correlated (R2 = 0.26). B. Proteins that change in abundance in a previous study of the aging proteome in C. elegans [19] generally change in the concordant direction in this study. The data are displayed as heat maps showing average log2 ratios of (old/young) expression of proteins that increased or decreased in abundance at least 1.5-fold between day 1 and day 12 in the Walther et al study (DMW) that were also present in our study (SMZ). 72% of proteins that increased in abundance in the Walther et al study also increased in our study (p<10−12 by binomial test), and 84% of the proteins that decreased in abundance in the Walther et al study also decreased in our study (p<10−8 by binomial test). However, the aging fold changes of proteins in the two studies were only modestly correlated (R2 = 0.33). C. Proteins that were previously found to be age-insoluble [17, 18] do not generally change in abundance in this study. The bar graphs show the fraction of proteins that were determined to be age-insoluble in two previous studies for all 1796 proteins covered in this study (all), the proteins that significantly increased in abundance (up), or proteins that significantly decreased (down). There was no significant enrichment for age- [file pgen.1005725.s001.tif]

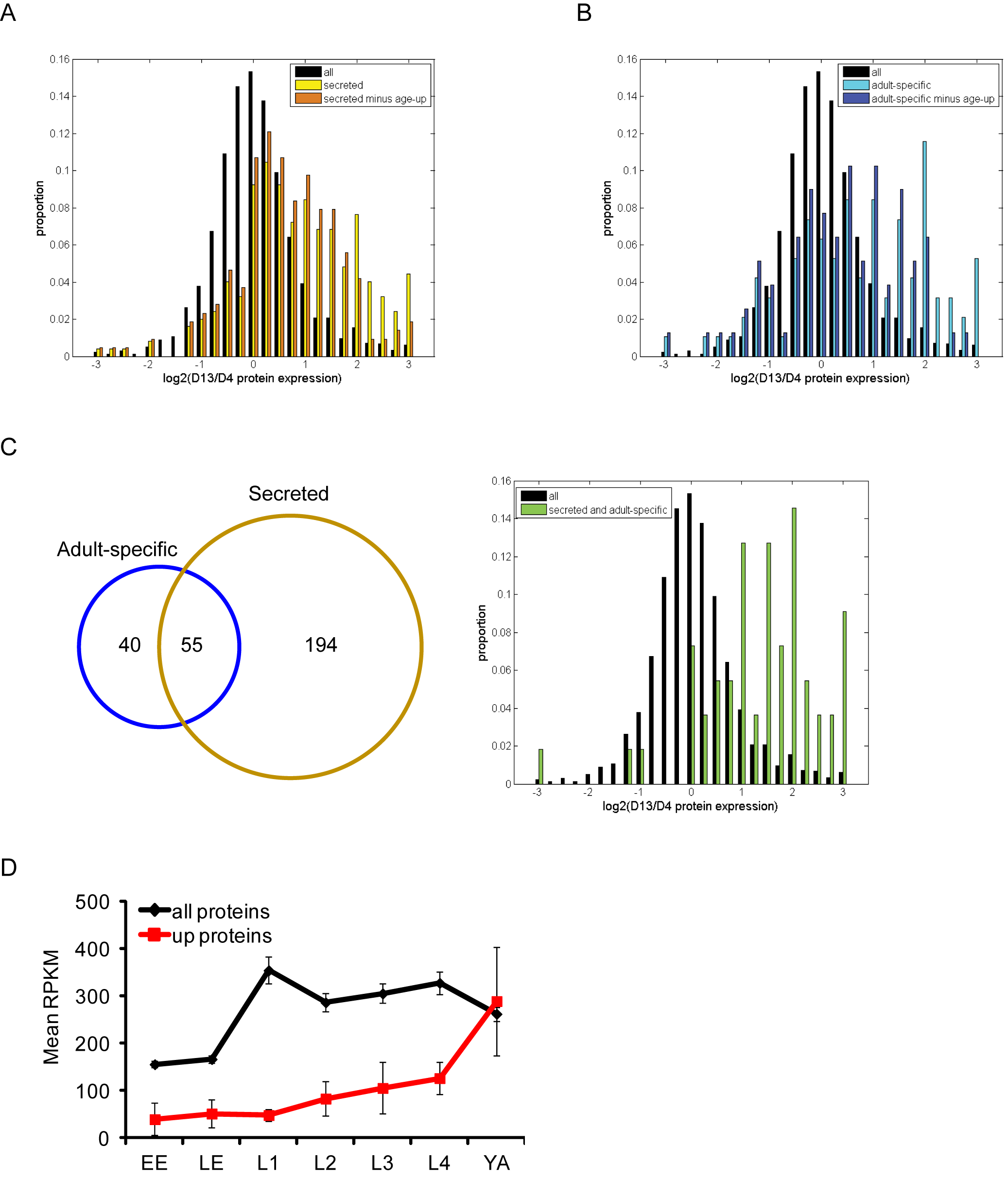

Supplement: S2 Fig — A. Secreted proteins tend to increase in abundance with age. The histogram shows the distribution of aging fold changes of all 1796 proteins covered in our data (black), the 249 proteins that are predicted to be secreted (yellow) and were covered in our data, and the same set of secreted proteins minus the 34 proteins that also significantly increase in abundance with age (orange). Secreted proteins are significantly shifted towards increasing in abundance with age (p < 10−27 by Kolmogorov-Smirnov test), even when the 34 proteins that are both significantly increased and secreted are removed (p < 10−17 by Kolmogorov-Smirnov test). B. Adult-specific proteins tend to increase in abundance with age. The histogram shows the distribution of aging fold changes of all 1796 proteins covered in our data (black), the 95 proteins whose transcripts are expressed specifically in adults (light blue), and the same set of adult-specific proteins minus the 17 proteins that also significantly increase in abundance with age (dark blue). Adult-specific proteins are significantly shifted towards increasing in abundance (p < 10−10 by Kolmogorov-Smirnov test), even when the 17 proteins that are both significantly increased in abundance with age and adult-specific are removed (p < 10−4 by Kolmogorov-Smirnov test). C. The Venn diagram (left) shows the overlap between the 95 proteins whose transcripts are expressed specifically in adults and the 249 proteins that are predicted to be secreted. 55 proteins are both adult-specific and secreted (a 4-fold enrichment over expectation, p<10−24 by Fisher’s exact test). The histogram (right) shows the distribution of aging fold changes of these 55 proteins (green) compared to all 1796 proteins covered in our experiment (black). The proteins that are both secreted and expressed specifically in adults are strongly shifted towards increasing in abundance with age (p < 10−18 by Kolmogorov-Smirnov test). D. Proteins that increase in abundance with age hav [file pgen.1005725.s002.tif]

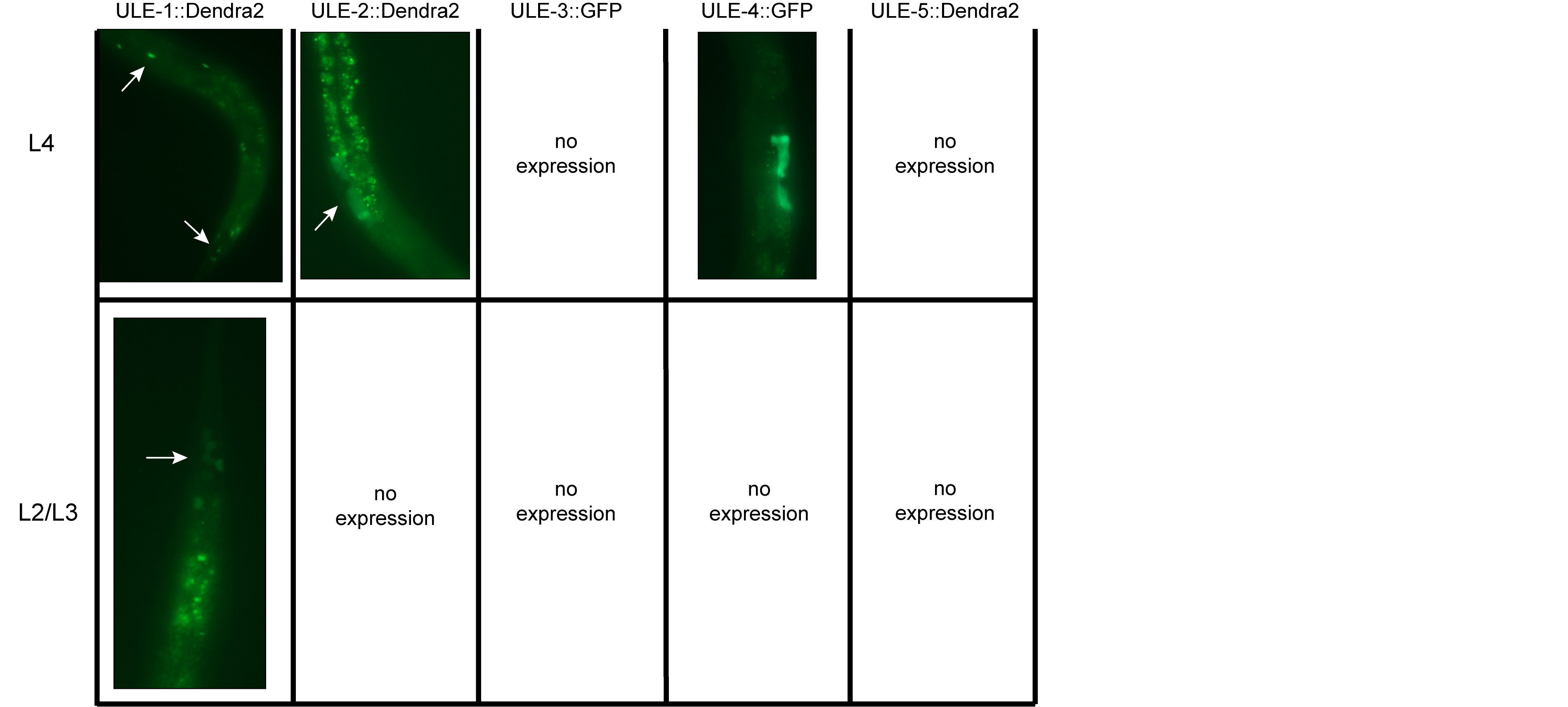

Supplement: S3 Fig — Representative images of the five uterine protein reporters in early larvae (L2/L3), L4 larvae, and young adult males (day 1–2 of adulthood). White arrows indicate fluorescent protein expression. “No expression” means that no fluorescent protein expression was visible at 63x magnification. Proteins were tagged at the C-terminus with eGFP (ULE-3, ULE-4) or Dendra2 (ULE-1, ULE-2, ULE-5). At least 20 worms of each reporter at each stage were examined. (TIF) [file pgen.1005725.s003.tif]

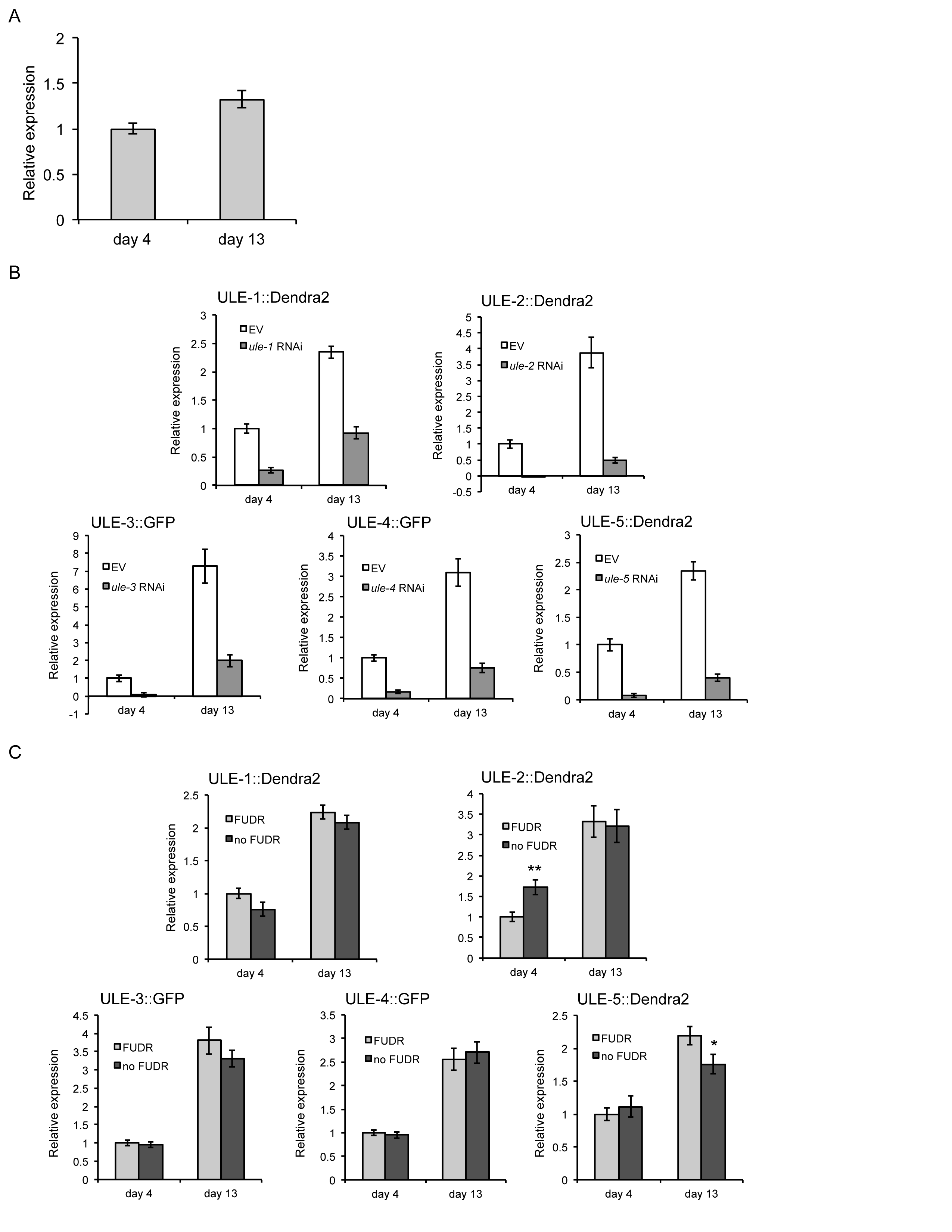

Supplement: S4 Fig — A. Autofluorescence increases 30% between day 4 and day 13 in non-transgenic N2 control animals. The data are represented as mean fluorescent intensity at day 13 relative to mean fluorescent intensity at day 4 (n = ~20 worms in each condition). Error bars are ± SEM. B. RNAi against each ULE gene reduces expression of its reporter at least 3-fold at both day 4 and day 13 for all five lines (p<0.001 by Student’s t-test for RNAi vs. control treated worms for all lines at both timepoints). Worms expressing uterine protein reporters were placed on empty vector control or the ULE gene RNAi as L1 larvae and imaged as day 4 and day 13 adults (n = ~20 worms for each line at each timepoint). The data are shown as mean fluorescent intensity minus the mean expression in a non-transgenic control worms fed empty vector RNAi. Error bars are ± SEM. C. Effect of FUDR on uterine protein reporter expression. Worms expressing uterine protein reporters were grown in the presence or absence of 30 mM FUDR starting at day 1 of adulthood and imaged at day 4 and day 13. Expression of ULE-2::Dendra2 increased 70% at day 4 in worms grown without FUDR, while ULE-5::Dendra2 expression decreased 20% at day 13 in worms grown without FUDR. The data are represented as mean fluorescent intensity relative to that of day 4 FUDR treated animals (n = ~20 worms in each condition). Error bars are ± SEM. *p<0.05 and **p<0.01 by Student’s t-test, comparing FUDR to no FUDR at each timepoint. (TIF) [file pgen.1005725.s004.tif]

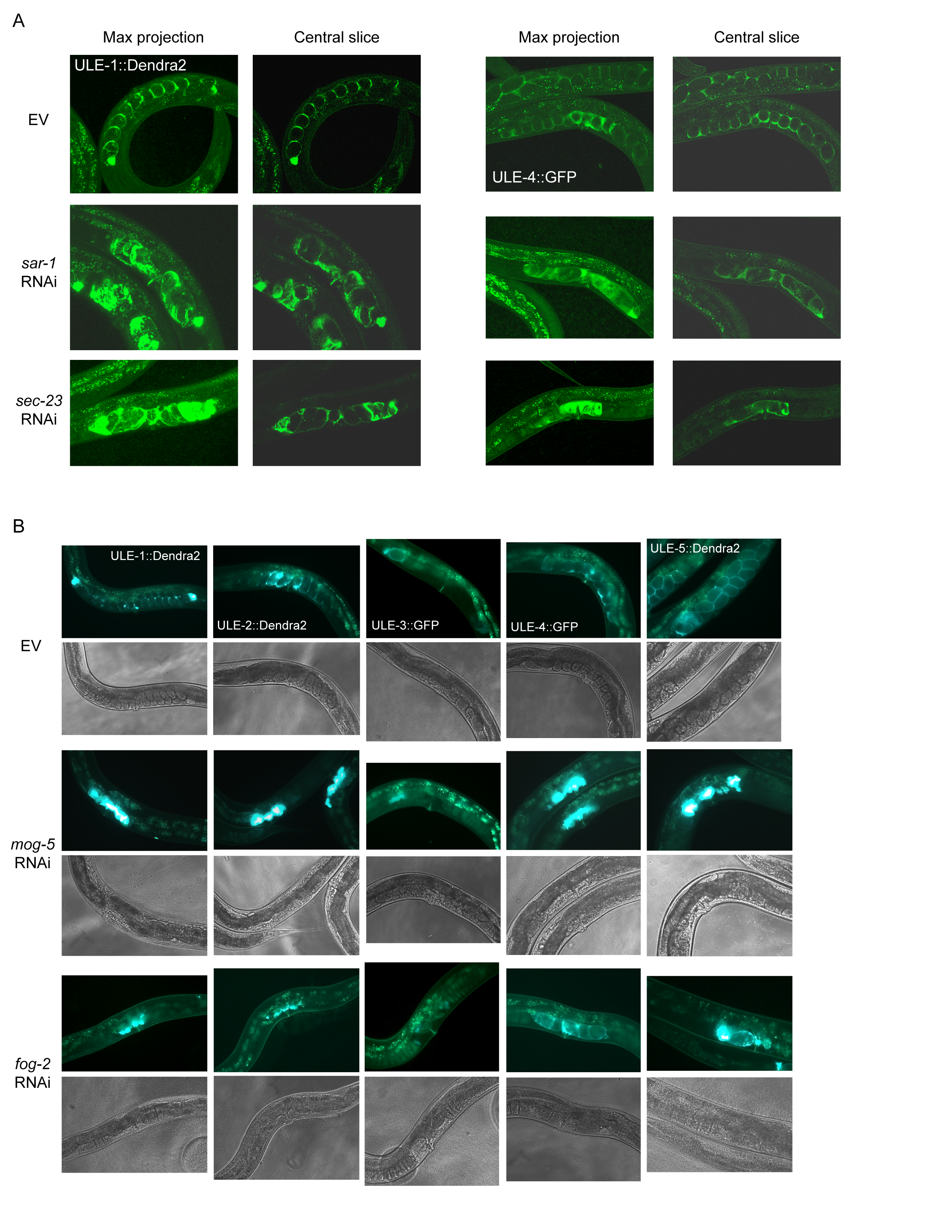

Supplement: S5 Fig — A. Uterine proteins are produced by the uterine cells (shown in Fig 4A and this figure) or the cells of the spermatheca (shown in Fig 4A). Worms expressing uterine protein reporters were placed on empty vector control RNAi or RNAi against sar-1 or sec-23 at L4 and imaged 24 hours later. Representative images of worms expressing ULE-1::Dendra2 (left) or ULE-4::GFP (right) are shown as a maximal projection of all images in the z-stack or the central image of the stack. B. Oocytes and sperm are not required for uterine protein reporter expression. Representative fluorescent (top) and DIC (bottom) images of day 1 adult worms expressing uterine protein reporters that were treated with empty vector, mog-5, or fog-2 RNAi starting as embryos. (TIF) [file pgen.1005725.s005.tif]

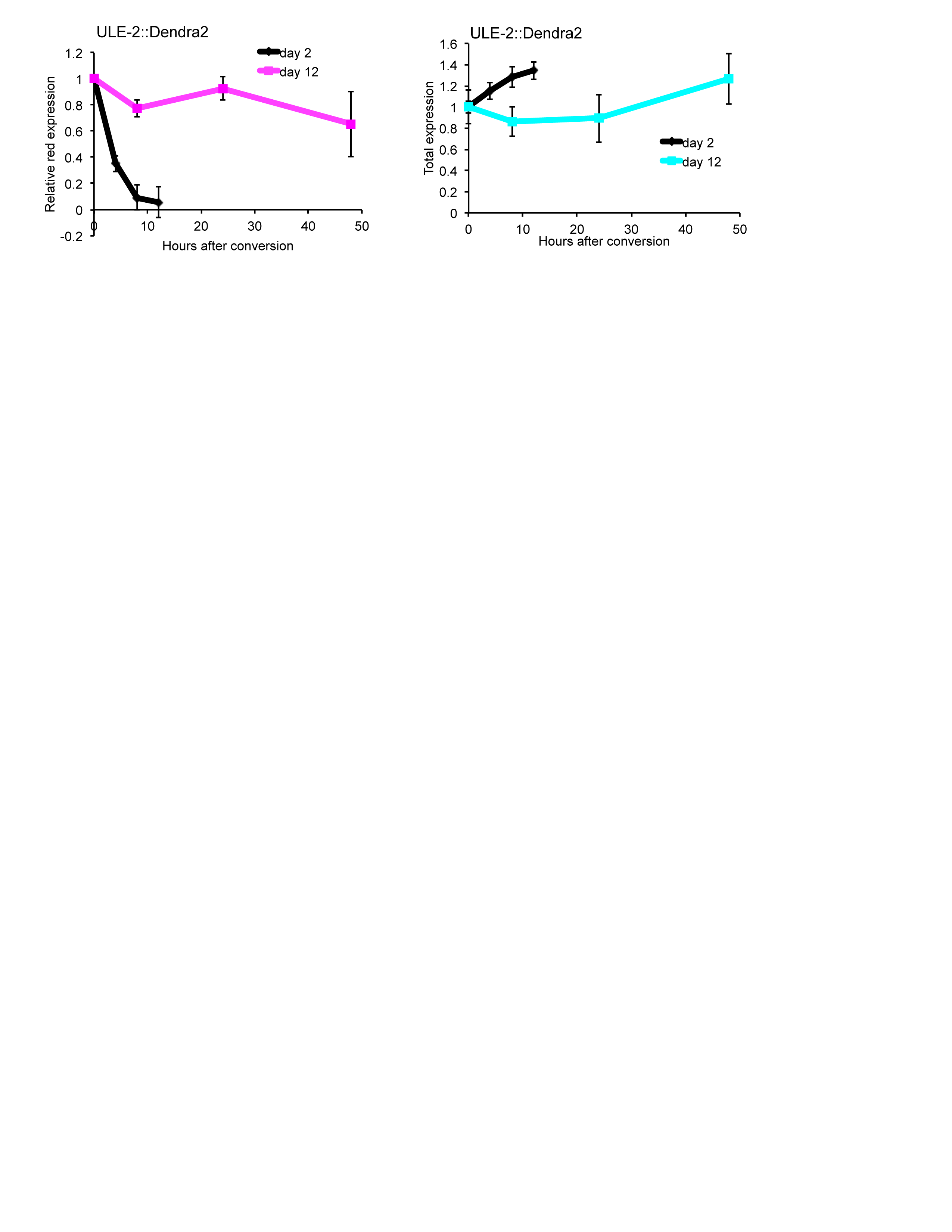

Supplement: S6 Fig — Biological replicate experiment of the measurement of protein dynamics of ULE-2::Dendra2 shown in Fig 5. Left: Dendra2 was photoconverted to the red form of the protein at time 0 in day 2 and day 12 adult worms expressing ULE-2:Dendra2. The same worms were re-imaged at subsequent time points to monitor the decay of red protein expression. The red expression values for each worm were background subtracted using the average red expression in unconverted worms as background. The data are represented as the average background subtracted red expression at each time point relative to the background subtracted red expression of the same worm at time 0 (n = 12 converted and 17 unconverted worms at day 2, and 16 converted and 16 unconverted worms at day 12). Right: The average green fluorescent intensity of unconverted worms at each time point relative to that of the same worms at time 0 (n = X). Error bars are ± SEM. (TIF) [file pgen.1005725.s006.tif]

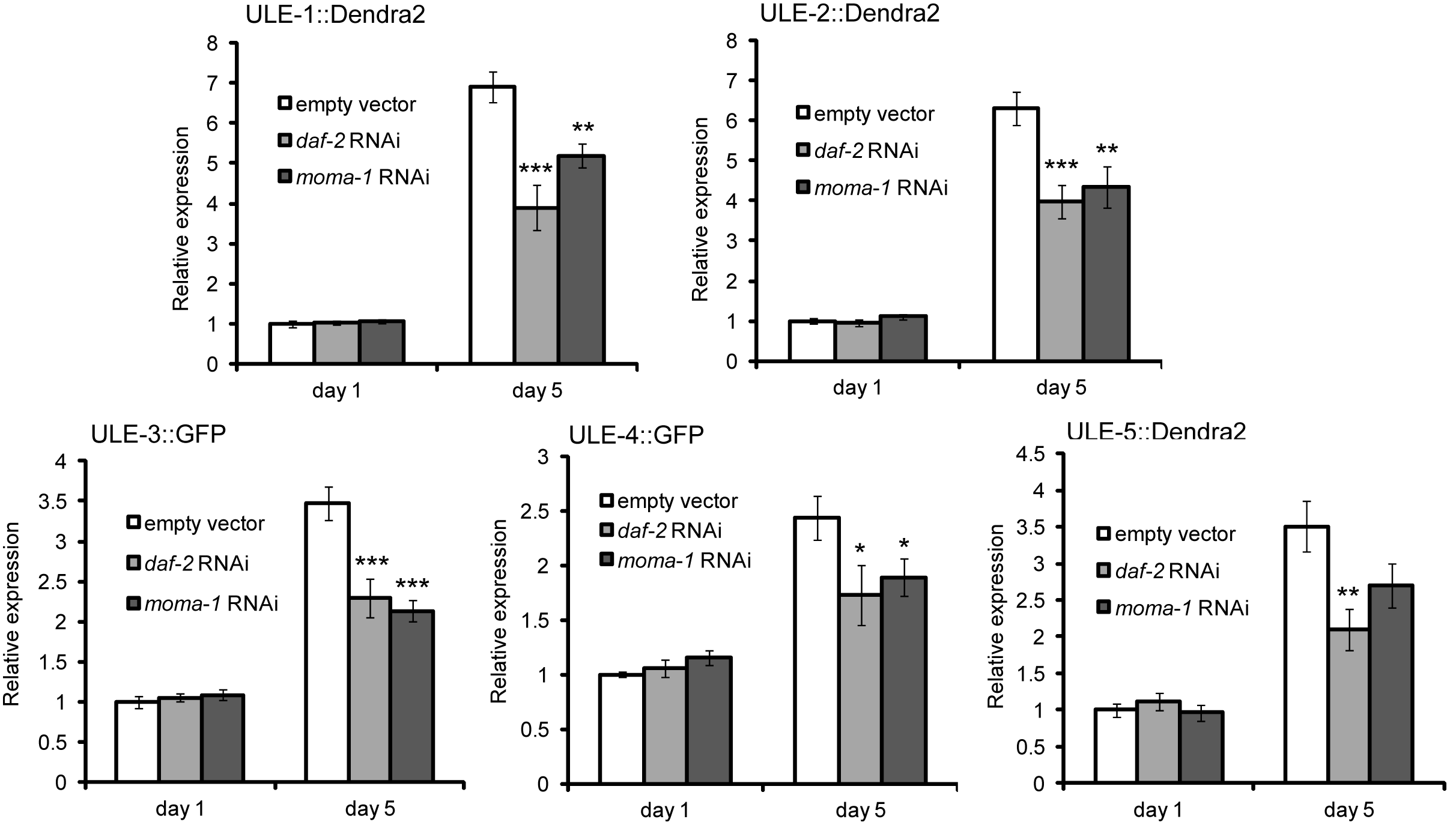

Supplement: S7 Fig — Worms expressing uterine protein reporters were placed on empty vector control RNAi or RNAi against daf-2 or moma-1 as L1 stage larvae and imaged on day 1 or day 5 of adulthood. The data are represented as mean fluorescent intensity relative to that of day 1 empty vector control treated animals (n = ~20 worms in each condition). Error bars are ± SEM. *p<0.05, **p<0.01, and ***p<0.001 by Student’s t-test, comparing day 5 empty vector control worms to day 5 daf-2 or moma-1 RNAi worms. There is no significant effect of daf-2 or moma-1 RNAi on the expression of any of the five reporters at day 1. (TIF) [file pgen.1005725.s007.tif]

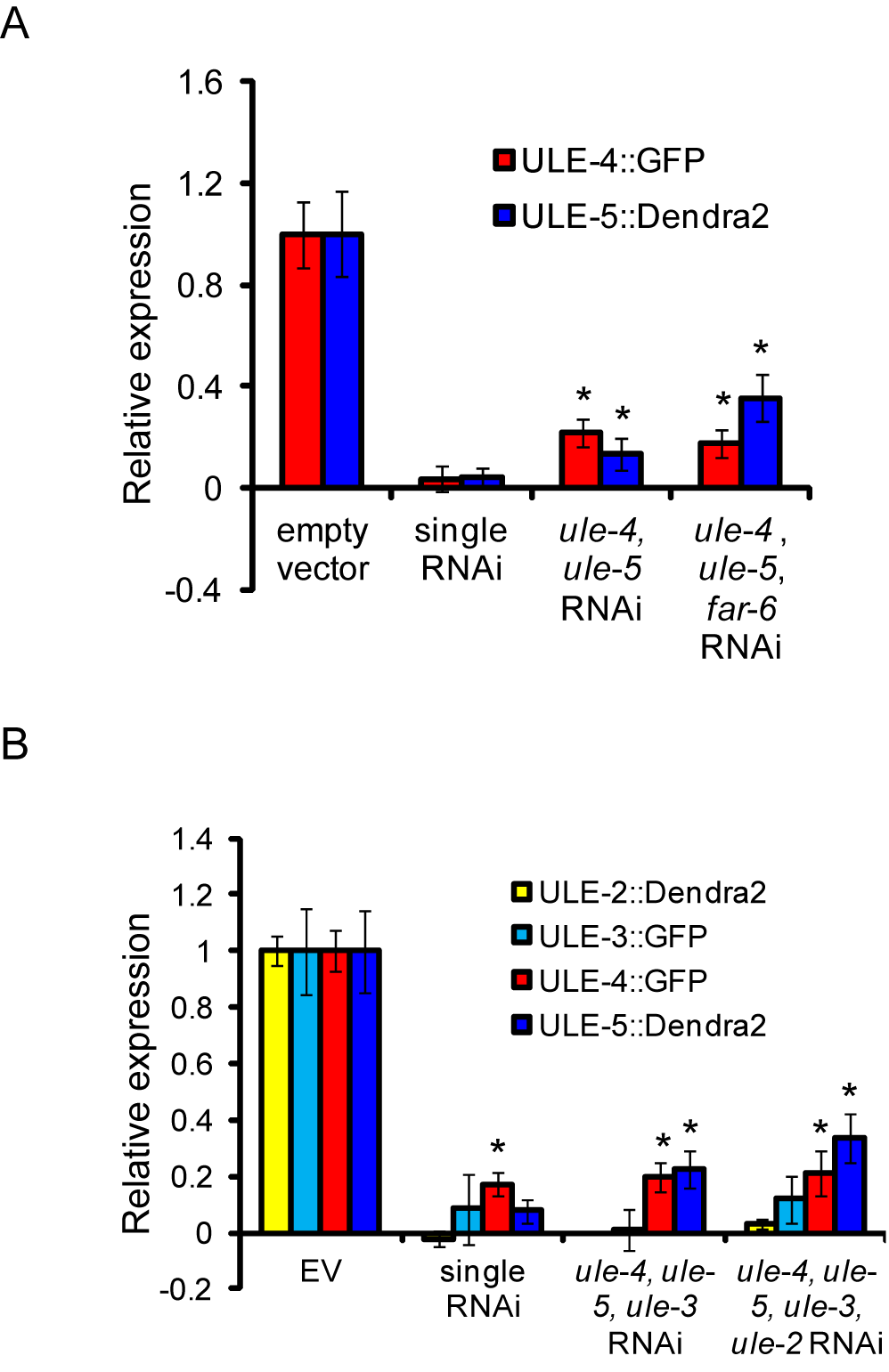

Supplement: S8 Fig — A. RNAi knockdown of ule-4 and ule-5 by mixed RNAi treatment is effective. Wild-type worms and worms expressing ULE-4::GFP or ULE-5::Dendra2 were placed on empty vector control RNAi, either ule-4 RNAi or ule-5 RNAi (single RNAi), an equal mixture of ule-4 RNAi and ule-5 RNAi, or an equal mixture of ule-4, ule-5, and far-6 RNAi as L1 larvae and imaged as day 3 adults. Single RNAi treatment reduced the level of both reporters to that of non-transgenic worms. Double and triple RNAi treatments substantially reduced the expression of both reporters but had slightly reduced effectiveness (*p<0.05 by Student’s t-test comparing RNAi treated worms to a non-transgenic control). The data are shown as mean fluorescent intensity minus the mean expression in a non-transgenic control worms fed empty vector RNAi. Error bars are ± SEM. B. RNAi knockdown of ule-4, ule-5, ule-3, and ule-2 by mixed RNAi treatment is effective. Wild-type worms and worms expressing uterine protein reporters were placed on empty vector control RNAi, RNAi against just the reporter gene (single RNAi), an equal mixture of ule-4 RNAi, ule-5 RNAi, and ule-3 RNAi, or an equal mixture of ule-4 RNAi, ule-5 RNAi, ule-3 RNAi, and ule-2 RNAi as L1 larvae and imaged as day 3 adults. Single RNAi treatment reduced the level of all reporters to that of non-transgenic worms. Triple and quadruple RNAi treatments reduced the expression of all reporters but had reduced effectiveness (*p<0.05 by Student’s t-test comparing RNAi treated worms to a non-transgenic control). The data are shown as mean fluorescent intensity minus the mean expression in a non-transgenic control worms fed empty vector RNAi. Error bars are ± SEM. (TIF) [file pgen.1005725.s008.tif]

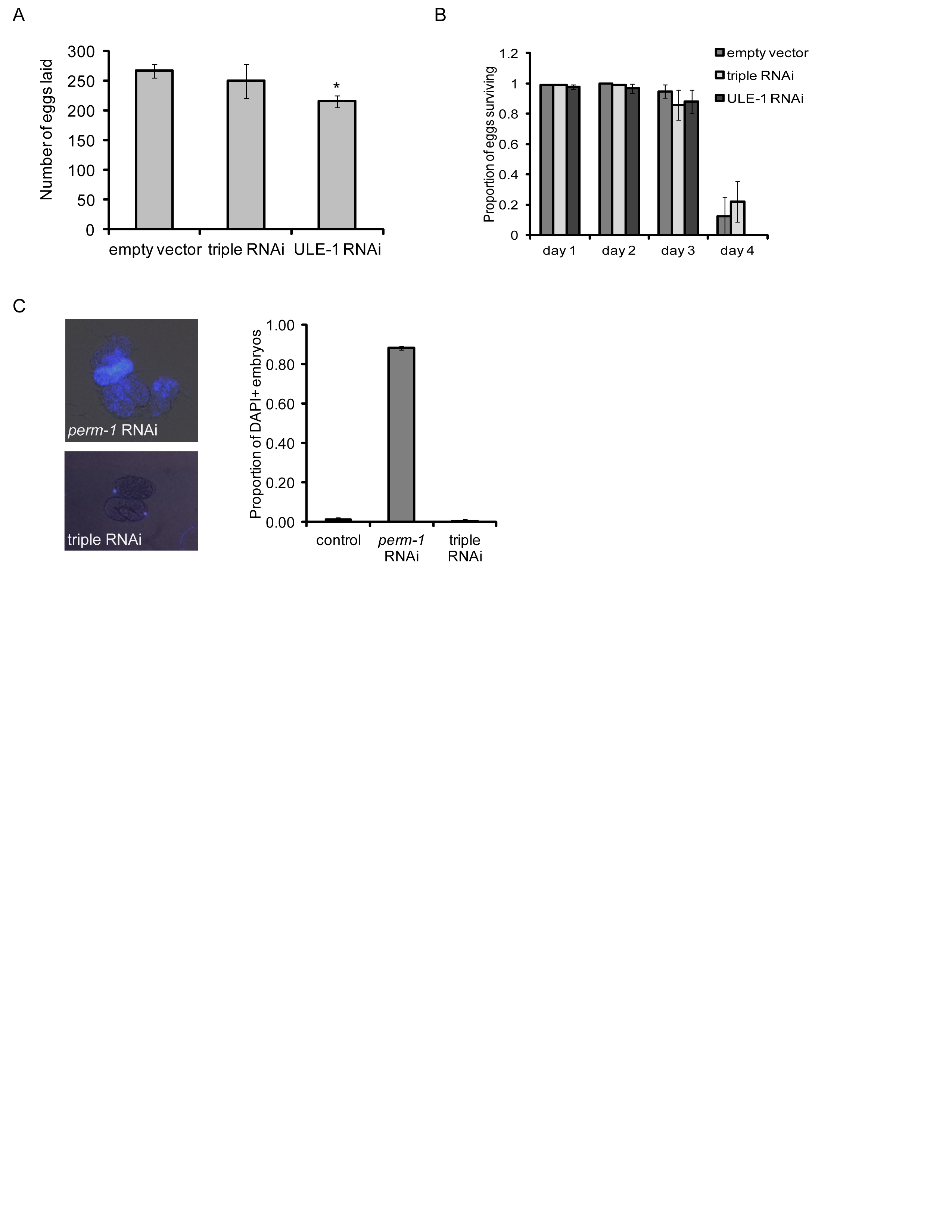

Supplement: S9 Fig — A. Knockdown of ule-4, ule-5, and far-6 simultaneously does not affect the number of eggs laid, but knockdown of ule-1 produces a modest reduction in brood size. Worms in the first larval stage were placed on either empty vector control RNAi, an equal mixture of ule-4, ule-5, and far-6 triple RNAi, or ule-1 single RNAi and the number of eggs laid on days 1–4 of adulthood were counted. Data is shown as the mean total number of eggs laid per worm in the first four days of adulthood (n = ~10 worms per condition). Error bars are ± SEM. *p<0.05 by Student’s t-test compared to empty vector control worms. B. Single knockdown of ule-1 or triple knockdown of ule-4, ule-5, and far-6 does not affect survival of progeny. Worms in the first larval stage were placed on either empty vector control RNAi, an equal mixture of ule-4, ule-5, and far-6 triple RNAi, or ule-1 single RNAi and the number of eggs laid on days 1–4 of adulthood were counted. The percent of eggs that had hatched and developed at least to the fourth larval stage were counted 48 hours later. Data is shown as the mean proportion of eggs surviving that were laid on each of the first four days of adulthood (n = ~10 worms per condition). Error bars are ± SEM. C. Knockdown of ule-4, ule-5, and far-6 simultaneously by RNAi (triple RNAi) does not increase permeability of the eggshell to DAPI. RNAi against perm-1 was used as a positive control [68]. N2 worms were placed on the appropriate RNAi clone as L1 stage larvae and mixed stage embryos from the resulting day 1 adults were stained with 20 ng/mL DAPI. Representative composite fluorescent and DIC images (left) show DAPI positive perm-1 treated embryos and DAPI negative triple RNAi treated embryos. The graph (right) shows the percentage of embryos that were DAPI positive (n = 5 slides per condition, > 50 embryos per slide). Error bars are ± SEM. (TIF) [file pgen.1005725.s009.tif]
